# Decarboxylase activity of the non-starter lactic acid bacterium *Loigolactobacillus rennini* gives crack defects in Gouda cheese through the production of γ-aminobutyric acid

**DOI:** 10.1128/aem.01655-23

**Published:** 2024-01-17

**Authors:** Hannes Decadt, Louise Vermote, Cristian Díaz-Muñoz, Stefan Weckx, Luc De Vuyst

**Affiliations:** 1 Research Group of Industrial Microbiology and Food Biotechnology (IMDO), Faculty of Sciences and Bioengineering Sciences, Vrije Universiteit Brussel, Brussels, Belgium; Universita degli Studi di Napoli Federico II, Portici, Italy

**Keywords:** cheese with cracks, decarboxylation, metagenome-assembled genomes, brine, rennet, *Loigolactobacillus rennini*, *Tetragenococcus halophilus*, lactic acid bacteria, Gouda

## Abstract

Ten Gouda cheese wheels with an age of 31 weeks from six different batch productions were affected by a crack defect and displayed an unpleasant off-flavor. To unravel the causes of these defects, the concentrations of free amino acids, other organic acids, volatile organic compounds, and biogenic amines were quantified in zones around the cracks and in zones without cracks, and compared with those of similar Gouda cheeses without crack defect. The Gouda cheeses with cracks had a significantly different metabolome. The production of the non-proteinogenic amino acid γ-aminobutyric acid (GABA) could be unraveled as the key mechanism leading to crack formation, although the production of the biogenic amines cadaverine and putrescine contributed as well. High-throughput amplicon sequencing of the full-length 16S rRNA gene based on whole-community DNA revealed the presence of *Loigolactobacillus rennini* and *Tetragenococcus halophilus* as most abundant non-starter lactic acid bacteria in the zones with cracks. Shotgun metagenomic sequencing allowed to obtain a metagenome-assembled genome of both *Loil. rennini* and *T. halophilus*. However, only *Loil. rennini* contained genes necessary for the production of GABA, cadaverine, and putrescine. Metagenetics further revealed the brine and the rennet used during cheese manufacturing as the most plausible inoculation sources of both *Loil. rennini* and *T. halophilus*.

**IMPORTANCE:**

Crack defects in Gouda cheeses are still poorly understood, although they can lead to major economic losses in cheese companies. In this study, the bacterial cause of a crack defect in Gouda cheeses was identified, and the pathways involved in the crack formation were unraveled. Moreover, possible contamination sources were identified. The brine bath might be a major source of bacteria with the potential to deteriorate cheese quality, which suggests that cheese producers should regularly investigate the quality and microbial composition of their brines. This study illustrated how a multiphasic approach can understand and mitigate problems in a cheese company.

## INTRODUCTION

Cheese texture is one of the main characteristics that determines different cheese types and associated consumer expectations (1). One of these texture characteristics is the formation of gas openings or eyes, which can be desirable or not at all. Among the hard-type cheeses, eyes are unwanted in Cheddar cheese, moderately abundant and desirable in Gouda cheese, and highly desired in Emmental and other Swiss-type cheeses. The lack of eyes in Cheddar cheese is ascribed to the use of a homofermentative acidifier (*Lactococcus*) as the sole lactic acid bacteria (LAB) starter culture. Eye formation in Gouda cheeses is related to carbon dioxide production from the conversion of milk citrate, which is performed by citrate-consuming lactococci and leuconostocs; species of the latter LAB group also contribute to eye formation through their heterofermentative lactose metabolism (2, 3). In Swiss-type cheeses, the characteristic large eyes are mainly the result of a propionic acid fermentation carried out by a secondary *Propionibacterium* starter culture (mainly *Propionibacterium freudenreichii* subsp. *shermanii*), during which lactate of the primary fermentation performed by the LAB starter culture is converted into propionate, acetate, and carbon dioxide (4, 5).

The formation of eyes is comparable with a crystallization process in a supersaturated solution; when the carbon dioxide gas pressure exceeds saturation, openings start to form at nuclei formed by irregularities in the curd texture, such as small air bubbles, remaining whey pockets, or tiny apolar particles (e.g., dust) (2, 6–8). Apart from the extent of its bacterial production, the saturation of carbon dioxide depends on multiple abiotic factors; for instance, its solubility decreases with increasing temperature, pH, and salt percentage (9, 10). To obtain the desired eye shape, the cheese consistency should allow a viscous flow of the cheese body. When the fracture strain of the cheese body is low compared to the deformation force, slits or cracks are formed instead of eyes (11). Since the fracture strain decreases with increased proteolysis and moisture evaporation, gas produced at elevated ripening time is more likely to result in cracks (2, 11).

Whether eye formation is intended or not, all cheese types can suffer from undesired gas production. This irregularity is traditionally divided into early and late gas formation. In both cases, it concerns excessive carbon dioxide production and even hydrogen gas formation. Early gas production due to mixed-acid fermentation may occur within some days of the cheese manufacturing and is associated with the presence of coliforms, such as *Citrobacter*, *Enterobacter*, *Escherichia*, and *Serratia*, in the milk (12). These bacteria are generally considered as indicators of poor hygiene or the occurrence of mastitis (13). Since they do not survive pasteurization, they can only cause problems in raw milk cheeses or in cases of post-pasteurization contamination.

Late gas production can be related to species of at least three different microbial groups. First, spore-forming butyric acid bacteria, such as *Clostridium tyrobutyricum,* can ferment lactate to butyrate, which is accompanied with carbon dioxide and hydrogen gas production (14). Spores of these bacteria can survive pasteurization and germinate in the anaerobic cheese environment during ripening. Especially semi-hard and hard, brine-salted cheeses, such as Gouda cheeses, are sensitive for clostridial growth due to the slow diffusion of the inhibitory salt into the cheese core (15–17). Second, *Propionibacterium* strains that are used as secondary starter cultures for the production of Swiss-type cheeses, or that are present in other cheese productions due to cross-contamination, can co-ferment aspartate and lactate and thus produce much more carbon dioxide gas compared to common propionic acid fermentation (10, 18). Third, different species of the *Lactobacillaceae* have been reported as causes of gas defects. In Cheddar cheese, *Streptococcus thermophilus* as part of the primary starter culture only metabolizes the glucose moiety of lactose. This leads to an excess of galactose that can be used by heterofermentative non-starter lactic acid bacteria (NSLAB), resulting in carbon dioxide production (19, 20). In Gouda-type cheeses, lactose and galactose are completely consumed within the first weeks of ripening (2, 21, 22). Hence, co-fermentation cannot be the cause of excessive gas production in this case. Yet, crack formation does occur in Gouda-type cheese productions (16). Several decades ago, *Loigolactobacillus bifermentans* as NSLAB inhabitant has been reported as a cause of cracks in Gouda cheeses (23). Later on, *Loigolactobacillus rennini* that was first isolated from cheese rennets has been indicated as undesirable gas producer in Gouda cheeses (24, 25). Although the genus name *Loigolactobacillus* refers to this spoilage (loigos is Greek for destruction), the pathways that lay at the basis of the concomitant crack formation have never been clarified. Afterward, a *Paucilactobacillus* species [formerly described as “*Lactobacillus danicus*,” which has never officially been recognized as species but seems to be closely related to *Paucilactobacillus oligofermentans* (26)] has been reported as late gas former in a Danish Gouda-type cheese, but the mechanism behind this gas production has not been unraveled either (27). However, it is likely to assume that undesirable gas formation, in particular carbon dioxide production, by these diverse members of the *Lactobacillaceae* in Gouda-type cheeses has to be ascribed to decarboxylase activities. Gouda cheese is generally made from pasteurized cow’s milk (fat content of 40%–50%) with a mesophilic starter culture and calf rennet. The curd formed is washed with warm water, molded, pressed, and brined for 2–4 days, depending on size and mass. Maturation takes place under controlled conditions of temperature and relative humidity for 4 weeks to 2 years and results in semi-hard, hard, or extra hard cheeses with a salt content of 2.5%–3.5% on dry matter (2, 16). It is obvious that, as Gouda cheese ages, the moisture content decreases, and the salt content becomes more concentrated, resulting in a higher salt-in-moisture content (28).

The aim of the present study was to identify not only the microbial species but also the underlying metabolic pathways leading to crack defects because of late gas production in Gouda cheese. Hereto, the overall metabolome and microbiota of 10 Gouda cheese wheels with an age of 31 weeks and coming from 6 different batch productions with crack defects were assessed and compared with earlier data of Gouda cheese wheels without crack defects, all cheese wheels being manufactured in the same European dairy company (21). Additionally, shotgun metagenomic sequencing was applied to gain insights into the genetic potential of the cheese microbiota related to gas production. Finally, this study aimed to identify possible inoculation sources of the microbial species related to these crack defects, more specifically the brine and rennet used, both culture dependently and culture independently.

## RESULTS

### Cheeses

**Fig 1:**
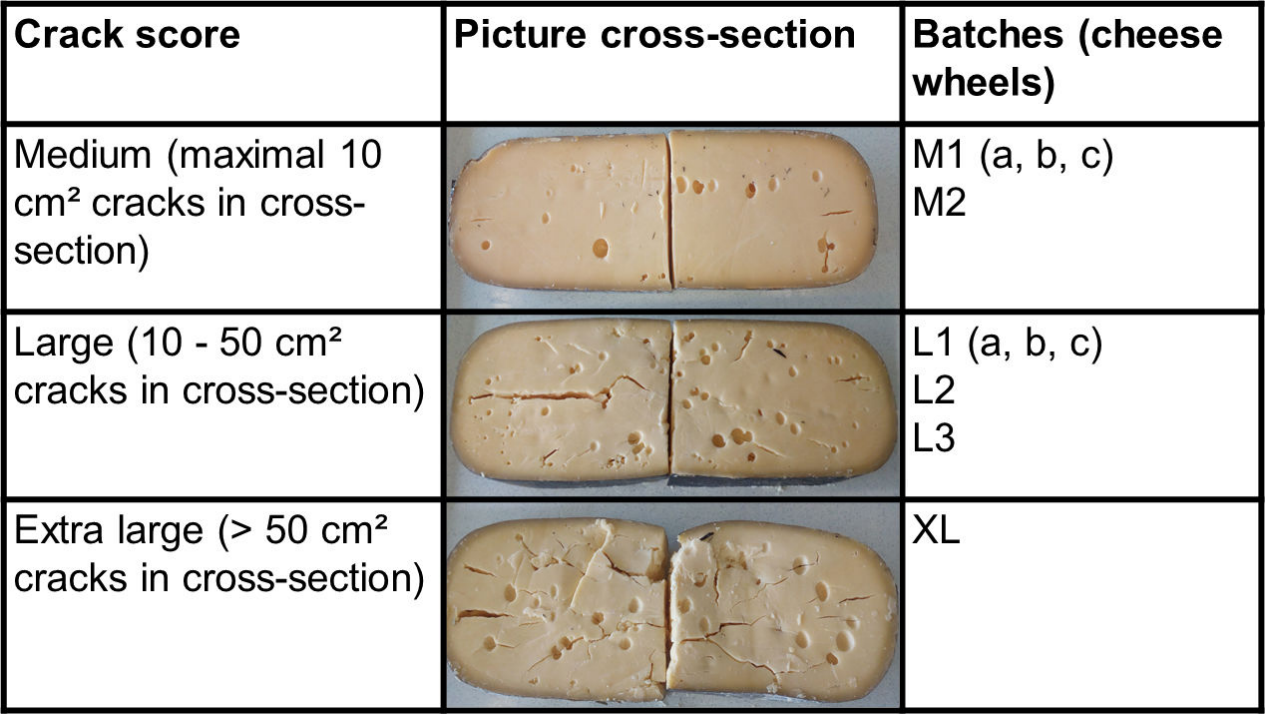
Classification, cross-section, and indications of Gouda cheese wheels with an age of 31 weeks and a crack defect produced in a European dairy factory.

Ten Gouda cheese wheels with an age of 31 weeks, coming from different batch productions, and that were all affected by crack formation and off-flavors were investigated on metabolic and microbiological level to reveal the causes of the cheese defects. Hereto, the zones around the cracks and the zones without visible cracks were investigated separately (Fig. 1).

### Physicochemical parameters

**Fig 2:**
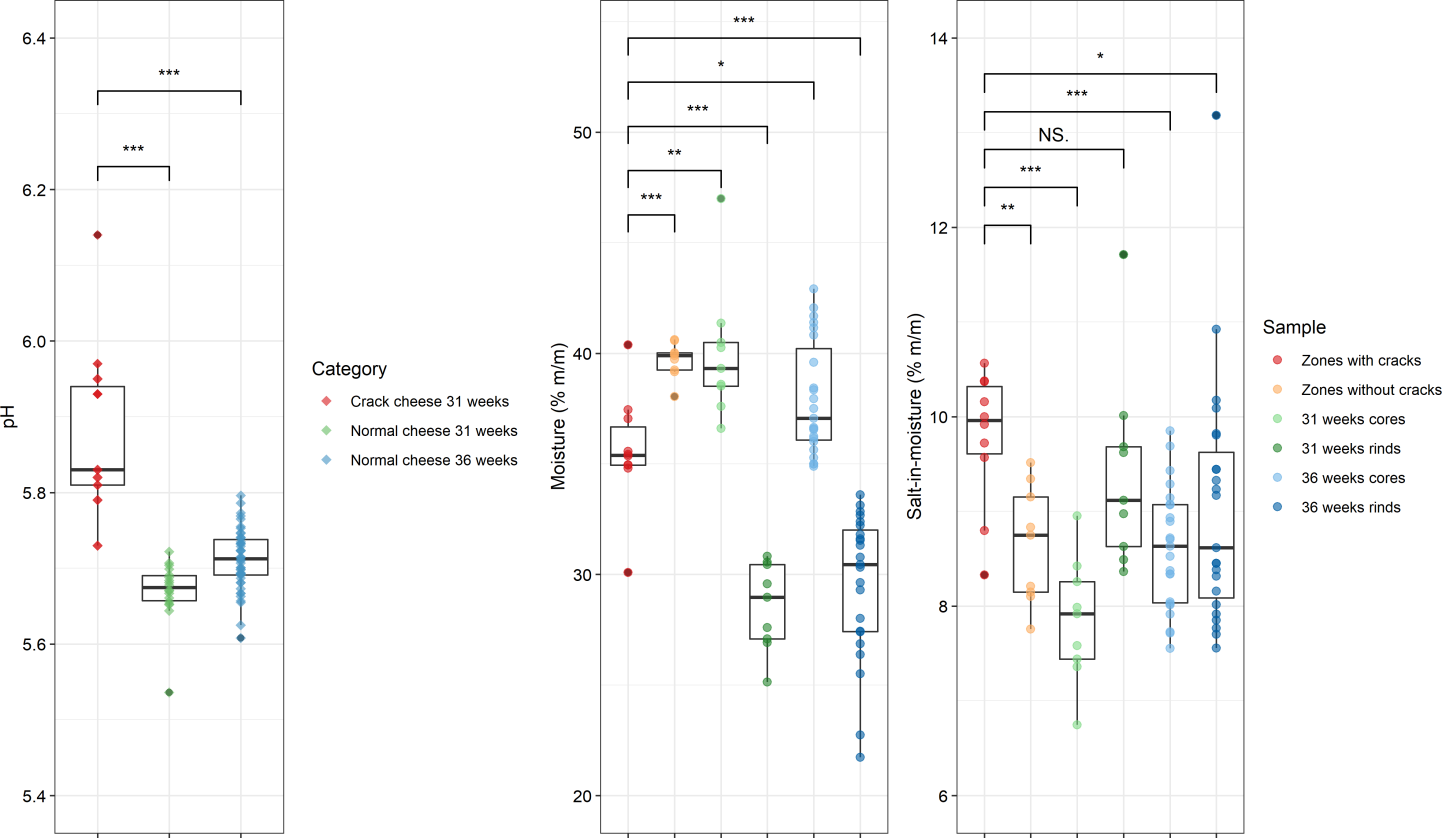
Physicochemical parameters of Gouda cheeses with cracks after 31 weeks of ripening compared to similar Gouda cheeses without cracks after 31 and 36 weeks of ripening (21). (**A**) pH of the Gouda cheeses with cracks (red) compared with similar Gouda cheeses without cracks after 31 and 36 weeks of ripening (green and blue, respectively). (**B**) Moisture (%, m/m) and salt in moisture (%, m/m) of the zones with cracks of the Gouda cheeses (red), the zones without cracks of the Gouda cheeses (orange), and the cores and rinds of similar Gouda cheeses without cracks after 31 weeks of ripening (light and dark green, respectively) and 36 weeks of ripening (light and dark blue, respectively). Significant differences between the zones with cracks of the Gouda cheeses and the other sampling areas are indicated with * (*P* < 0.05), ** (*P* < 0.01), *** (*P* < 0.001), or NS when no significant difference was found.

The zones with cracks of the Gouda cheese wheels examined had, on average, a pH of 5.89, a moisture content of 35.60% (m/m), and a salt-in-moisture content of 9.78% (m/m). These physicochemical parameters were compared with those of similar Gouda cheeses without cracks, made in the same dairy factory and ripened for 31 and 36 weeks, that have been reported previously (Fig. 2). The pH was significantly higher in the Gouda cheeses with cracks compared to those without cracks. The zones with cracks had a significantly lower moisture content compared to the zones without cracks and the cores of the similar Gouda cheeses without cracks ripened for 31 and 36 weeks but a significantly higher moisture content compared to the rinds of the latter cheeses. The salt-in-moisture content in the zones with cracks was significantly higher compared to the zones without cracks, the cores of the similar Gouda cheeses without cracks ripened for 31 weeks, and the cores and rinds of the latter cheeses ripened for 36 weeks.

### Metabolomics

**Fig 3:**
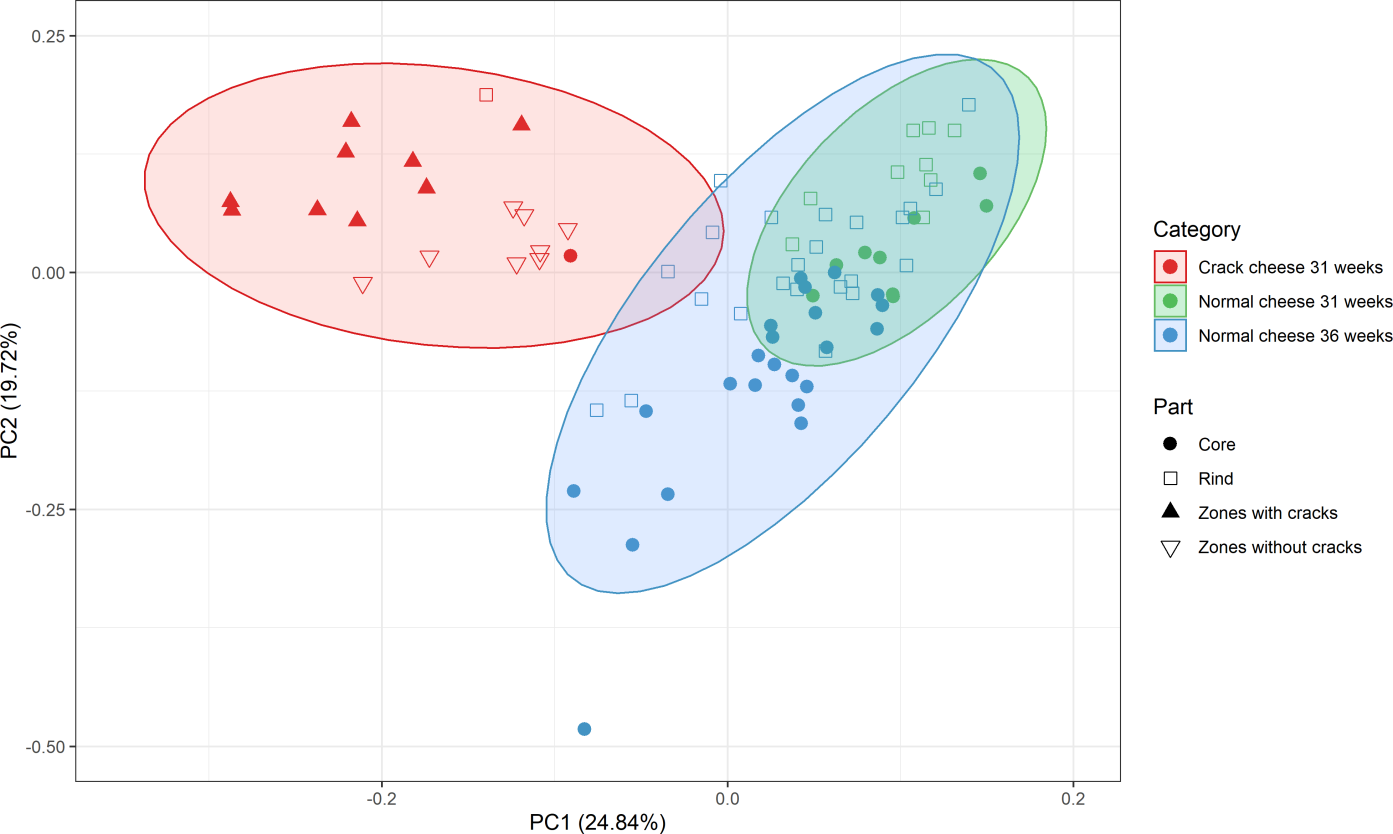
Principal component analysis of the concentrations of all metabolites measured in the Gouda cheeses with cracks after 31 weeks of ripening (red) and the similar Gouda cheeses without cracks after 31 and 36 weeks of ripening (green and blue, respectively). Zones with cracks, full triangles; zones without cracks, open triangles; cheese cores, circles; cheese rinds, squares.

A wide range of metabolites [amino acids, biogenic amines, short-chain fatty acids (SCFAs), other organic acids, and volatile organic compounds (VOCs)] was quantified. For a comprehensive understanding, the metabolite profile of the Gouda cheeses examined was compared with that of the similar Gouda cheeses without cracks ripened for 31 and 36 weeks. A principal component analysis distinguished both the zones with cracks and the zones without cracks of the Gouda cheeses examined from the cores and rinds of the similar Gouda cheeses without cracks ripened for 31 and 36 weeks (Fig. 3). From the Gouda cheese wheel M2 without visible cracks, the rind clustered with the zones with cracks, whereas the core clustered with the zones without cracks. Consequently, the rind sample of cheese M2 was considered as a crack-zones sample and the core sample as a zones-without-cracks sample.

**Fig 4:**
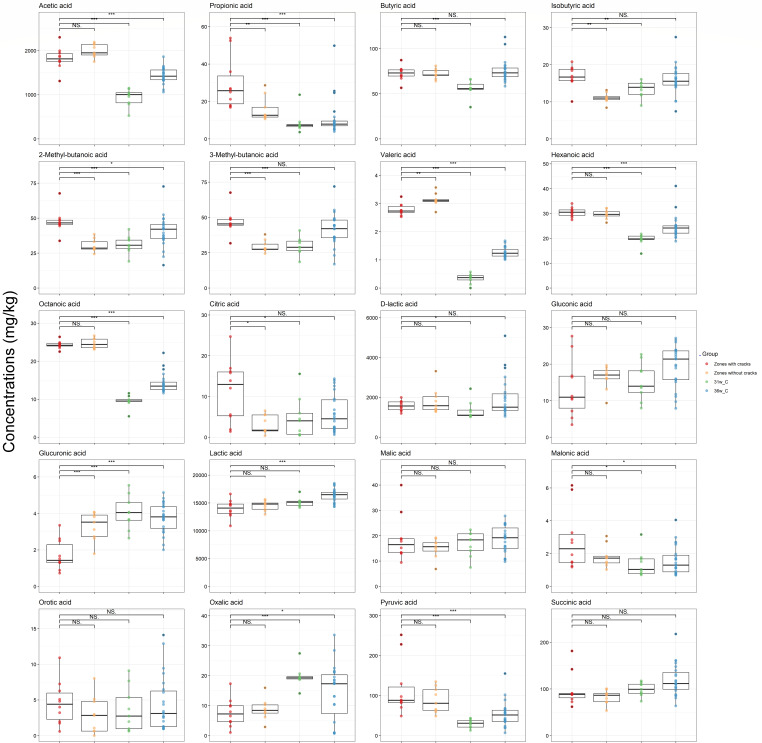
Boxplots of the concentrations of short-chain fatty acids and other organic acids in the zones with cracks of the 31-week Gouda cheeses (red), the zones without cracks of the 31-week Gouda cheeses (orange), and the cores of the similar Gouda cheeses without cracks after 31 and 36 weeks of ripening (green and blue, respectively). Significant differences between the concentrations in the zones with cracks of the Gouda cheeses and the other sampling areas are indicated with * (*P* < 0.05), ** (*P* < 0.01), *** (*P* < 0.001), or NS when no significant difference was found.

**Fig 5:**
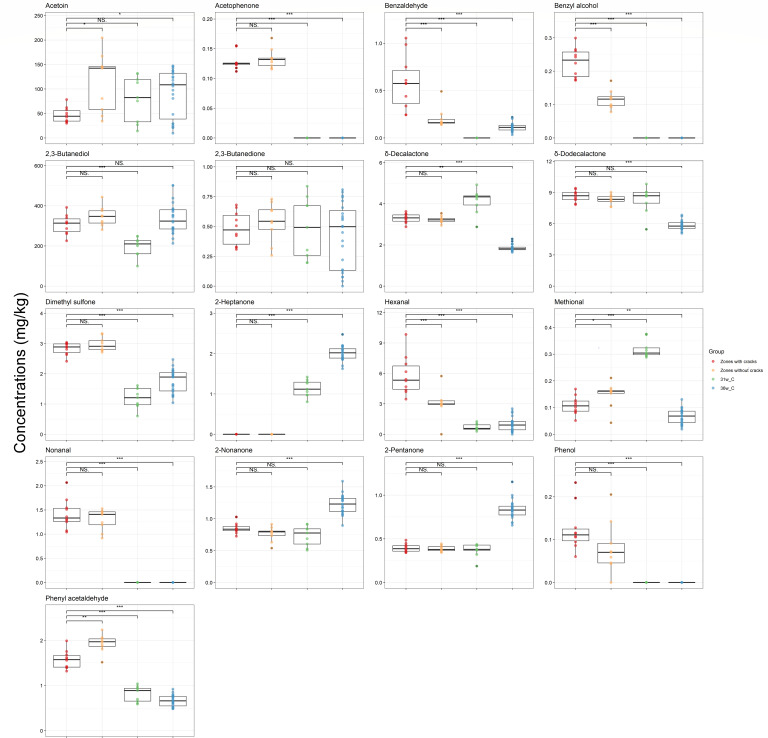
Boxplots of the concentrations of volatile organic compounds in the zones with cracks of the 31-week Gouda cheeses (red), the zones without cracks of the 31-week Gouda cheeses (orange), and the cores of the similar Gouda cheeses without cracks after 31 and 36 weeks of ripening (green and blue, respectively). Significant differences between the concentrations in the zones with cracks and the other sampling areas are indicated with * (*P* < 0.05), ** (*P* < 0.01), *** (*P* < 0.001), or NS when no significant difference was found.

**Fig 6:**
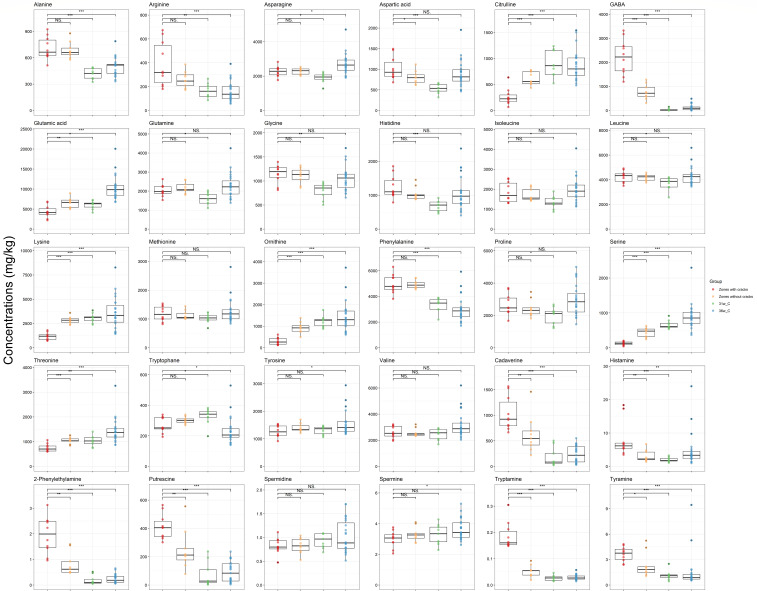
Boxplots of the concentrations of free amino acids and biogenic amines in the zones with cracks of the 31-week Gouda cheeses (red), the zones without cracks of the 31-week Gouda cheeses (orange), and the cores of the similar Gouda cheeses without cracks after 31 and 36 weeks of ripening (green and blue, respectively). Significant differences between the concentrations in the zones with cracks and the other sampling areas are indicated with * (*P* < 0.05), ** (*P* < 0.01), *** (*P* < 0.001), or NS when no significant difference was found.

The concentrations of 31 organic compounds differed significantly (*P* < 0.01) between the zones with cracks of the Gouda cheeses and the cores of the similar Gouda cheeses without cracks ripened for 31 and 36 weeks (Fig. 4 to 6). γ-Aminobutyric acid (GABA) had an average concentration of 2,213 mg/kg in the zones with cracks of the Gouda cheeses examined, which was 67 times higher compared to the concentrations found in the cores of the similar Gouda cheeses without cracks ripened for 31 weeks. Acetophenone, benzyl alcohol, nonanal, and phenol were only found in the Gouda cheeses with cracks. Additionally, concentrations of several amino acids (alanine, arginine, and phenylalanine), biogenic amines (cadaverine, histamine, 2-phenylethylamine, putrescine, tryptamine, and tyramine), SCFAs (acetic acid, propionic acid, valeric acid, hexanoic acid, and octanoic acid), organic acids (pyruvic acid), and VOCs (benzaldehyde, dimethyl sulfone, hexanal, and phenyl acetaldehyde) were significantly higher (*P* < 0.01) in the zones with cracks of the Gouda cheeses examined compared to the cores of the similar Gouda cheeses without cracks ripened for 31 and 36 weeks, whereas the concentrations of citrulline, lysine, ornithine, serine, threonine, glucuronic acid, and 2-heptanone were significantly lower (*P* < 0.01) in the zones with cracks of the Gouda cheeses examined compared to the cores of the similar Gouda cheeses without cracks ripened for 31 and 36 weeks. The concentrations of all these compounds in the zones without cracks of the Gouda cheeses examined were mostly in between the concentrations of these compounds in the zones with cracks of these cheeses and those of the similar Gouda cheeses ripened for 31 and 36 weeks. The concentrations of methional and δ-decalactone were higher (*P* < 0.01) in the similar Gouda cheeses without cracks ripened for 31 weeks and lower (*P* < 0.01) in those ripened for 36 weeks, compared to the zones with cracks of the Gouda cheeses examined, suggesting that these differences were related to the ripening time and that the Gouda cheeses with cracks might have an intermediate metabolite profile.

Some of the abundant organic compounds in the zones with cracks were the result of decarboxylation reactions, releasing carbon dioxide. In this way, the average concentrations of GABA, cadaverine, and putrescine in the zones with cracks equaled a released concentration of 21.46, 10.08, and 4.66 mmol of carbon dioxide per kilogram of cheese.

### Microbial counts

**Fig 7:**
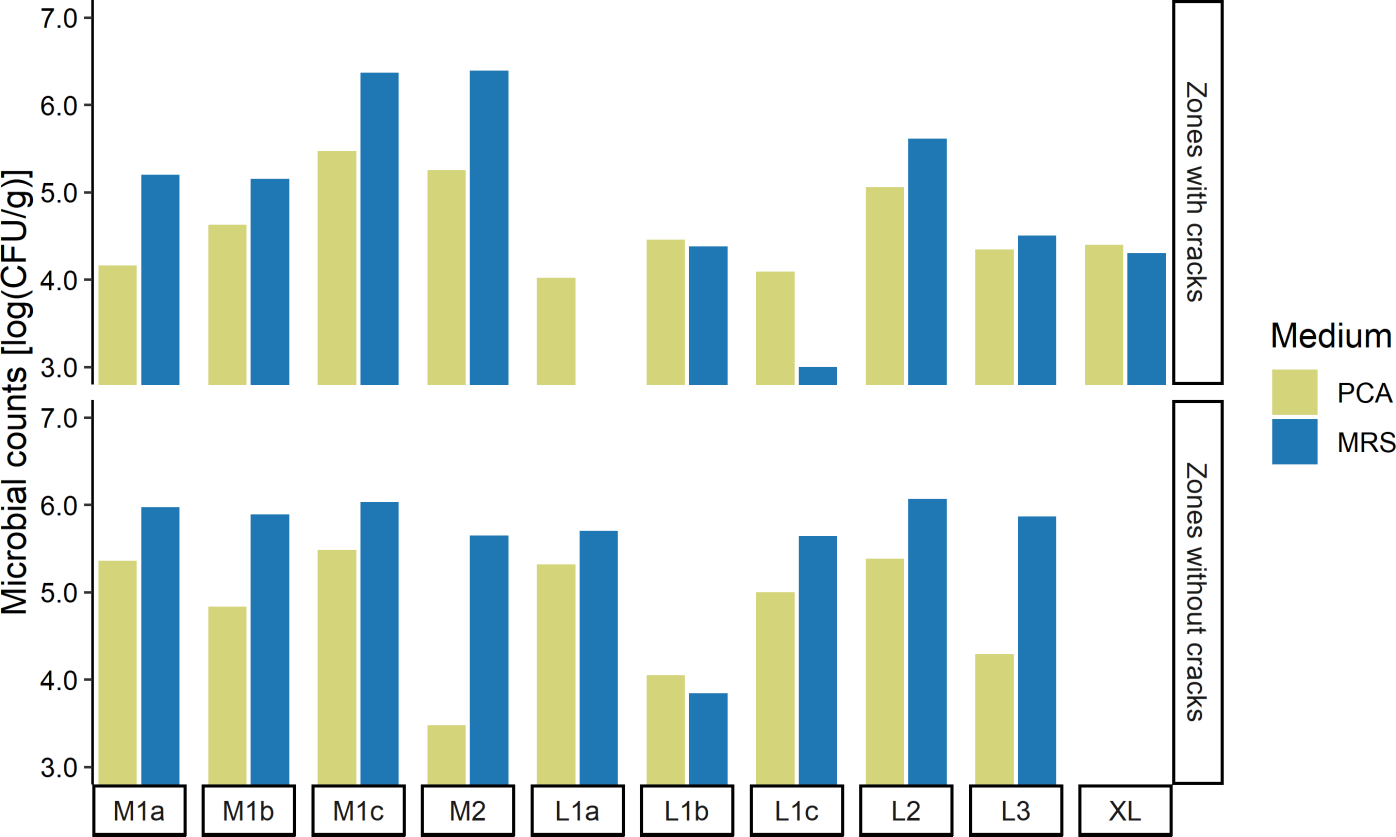
Microbial colony counts of the 10 Gouda cheese wheels with an age of 31 weeks examined on plate count agar (PCA) medium (green) and de Man-Rogosa-Sharpe (MRS) agar medium (blue) for the zones with cracks and the zones without cracks.

The plate count agar (PCA) counts were on average 4.6 log (CFU/g) in the zones with cracks of the Gouda cheeses examined and 4.8 log (CFU/g) in the zones without cracks, whereas the de Man-Rogosa-Sharpe (MRS) agar counts were on average 4.7 and 5.6 log (CFU/g) in the zones with cracks and the zones without cracks, respectively (Fig. 7). For the zones without cracks, the MRS counts were significantly higher than the PCA counts.

Amplicon-based high-throughput sequencing of the full-length 16S rRNA gene using DNA of cell suspensions from agar media washes mainly determined *Staphylococcus equorum* in the case of PCA medium and *Lacticaseibacillus paracasei* in the case of MRS agar medium, for both the zones with cracks and the zones without cracks of the Gouda cheeses examined. However, it is likely that not all viable microorganisms could be cultured.

### Metagenetics

**Fig 8:**
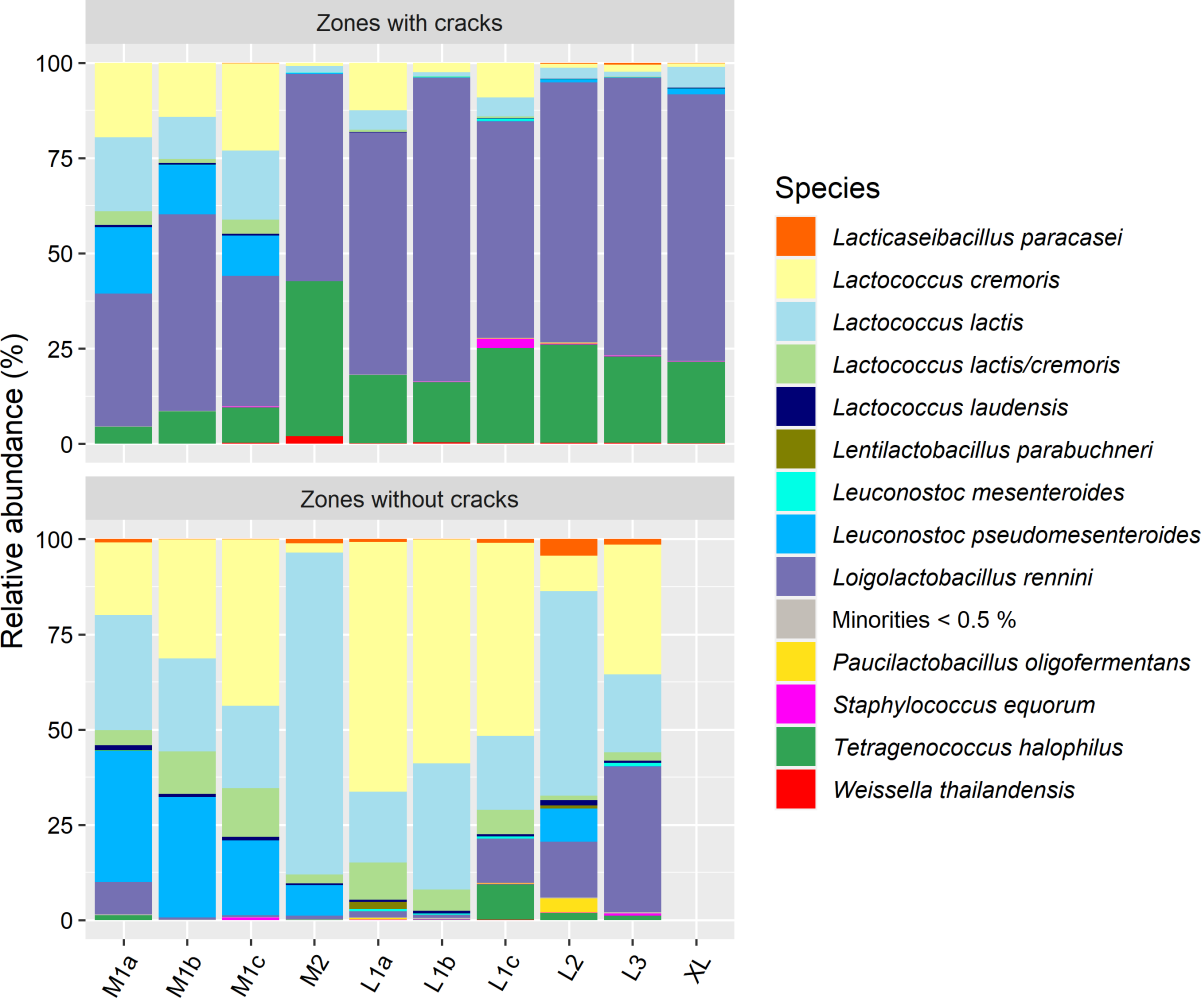
Taxonomic assessment of bacteria at species level, expressed as relative abundances based on amplicon sequence variants of the full-length 16S rRNA gene, for the zones with cracks and the zones without cracks of all 31-week Gouda cheese wheels investigated.

The most abundant bacterial species in the zones with cracks of the Gouda cheeses examined was *Loil. rennini*, followed by *Tetragenococcus halophilus* (Fig. 8). The zones without cracks harbored *Lactococcus cremoris* and *Lactococcus lactis* as the most prevailing species, followed by *Leuconostoc pseudomesenteroides*. Only a minor relative abundance of *Loil. rennini* and *T. halophilus* occurred in the zones without cracks of most Gouda cheeses. The *P*-value of the beta diversity concerning differences between the zones with cracks and the zones without cracks of the Gouda cheeses examined was 0.0001, demonstrating that the zones with cracks had a significantly different microbial profile compared to the zones without cracks.

In general, *Loil. rennini* and *T. halophilus* were positively correlated with the metabolites of which the concentrations were significantly higher in the zones with cracks of the Gouda cheeses examined (Table S1). Among these metabolites, the concentrations of benzyl alcohol, cadaverine, GABA, isobutyric acid, 2- and 3-methylbutanoic acid, and putrescine were higher correlated with *Loil. rennini* than with *T. halophilus*.

### Metagenome-assembled genomes

Metagenome-assembled genomes (MAGs) with high completion and low redundancy were obtained for the abundant bacteria *Loil. rennini* and *T. halophilus*, as well as for *Lacc. paracasei*, *Lactococcus laudensis*, and *Weissella thailandensis* (Table S2). Despite their high relative abundance in some Gouda cheese samples, no MAGs with acceptable completion and redundancy were retrieved for the starter LAB *Lc. cremoris*, *Lc. lactis*, *Leuconostoc mesenteroides*, and *Leuc. pseudomesenteroides*. Probably, the high interspecies similarity and the high intraspecies diversity hampered a proper assembly.

According to the supplier, no amino acid decarboxylases were present in the Gouda cheese starter cultures used. In contrast, different decarboxylase genes with the necessary transporters were found in the MAG of *Loil. rennini*, in particular genes for the production of GABA from glutamate (*gadC* and *gadB* as well as all eight subunits of the F_0_F_1_-ATPase system) and putrescine from ornithine (Table 1). Concerning the latter enzymatic conversion, two different ornithine decarboxylase genes (*odcI 1* and *odcI 2*) and a putrescine/ornithine antiporter gene (*potE*) were found in the MAG of *Loil. rennini*. These genes were comparable—on protein level—to those of *Ligilactobacillus saerimneri,* in that *odcI 1* shared 67% identity (and 81% similarity) with *odcI* of *Ligl. saerimneri*, whereas *odcI 2* shared 72% identity (and 85% similarity) with a lysine decarboxylase gene of *Ligl. saerimneri*, and the *potE* gene shared 69% identity (and 85% similarity) with that of *Ligl. saerimneri*. Also, a tryptophane decarboxylase gene was found in the MAG of *Loil. rennini*. The *Lc. laudensis* MAG also contained the *gadB* gene but not the antiporter gene *gadC*. None of these genes were present in the *T. halophilus* MAG.

**TABLE 1:** Genes encoding decarboxylases and related transporters found in the metagenome-assembled genomes retrieved from whole-community DNA isolated from the zones with cracks and the zones without cracks of 10 Gouda cheese wheels with an age of 31 weeks and a crack defect, for *Loigolactobacillus rennini, Lactococcus laudensis,* and *Lacticaseibacillus paracasei^a^*

Pathway	Gene	MAG	UniProt accession number	Identity (%)	Similarity (%)
Putrescine production	Ornithine carbamoyl-transferase	*Lc. laudensis*	Q5XAY4	66.17	76.95
		*Loil. rennini*	P99073	75.30	88.86
	Putrescine transporter	*Loil. rennini*	P0AAF1	60.32	79.14
	Inducible ornithine decarboxylase	*Lacc. paracasei*	P24169	39.97	58.83
		*Loil. rennini*	P43099	66.81	80.75
			P43099	60.78	77.05
γ-Aminobutyrate (GABA) production	Glutamate decarboxylase	*Lc. laudensis*	Q9CG20	56.52	80.94
		*Loil. rennini*	Q9CG20	53.42	70.64
	Glutamate/GABA antiporter	*Loil. rennini*	O30417	23.90	44.16
Serine degradation with ammonium production	L-serine dehydratase α chain	*Lacc. paracasei*	P33073	42.56	60.21
		*Lc. laudensis*	P33073	41.11	59.26
		*Loil. rennini*	P33073	41.16	61.22
	L-serine dehydratase β chain	*Lacc. paracasei*	P33074	35.38	52.82
		*Lc. laudensis*	P33074	37.31	56.48
		*Loil. rennini*	O34635	44.08	60.19
Tryptamine production	Tryptophan decarboxylase	*Loil. rennini*	A7B1V0	50.52	69.10

^*a*^ MAG, metagenome-assembled genome.

Finally, all contigs were screened for the histidine decarboxylase genes *hdcA* and *hdcB* before binning, but not any hit was found for both genes. Screening for the histidine/histamine antiporter gene *hdcP* of *T. halophilus* resulted in a 100% match. Inspection of the shared and unique KEGG functions of *Loil. rennini* and *T. halophilus* could not identify possible synergies between both species.

### Brines and rennets

Six brine samples (from two brine baths at locations A, B, and C) and seven commercial rennet samples (R1–R7) were examined.

### Physicochemical parameters of the brines

The pH values of brine 1 and brine 2 were on average 5.05 and 4.90, respectively, which were significantly different (Table S3). The temperature of both brines was on average 13.2°C, except for sample 2C. At location C, fresh cheeses entered the brine and increased the local temperature to 17.4°C. The NaCl content was exceptionally high in brine sample 1A, with 259 g/L, whereas it was on average 204 g/L in the other brine samples.

### Metabolites of the brines

The brines contained several carbohydrates, organic acids, SCFAs, amino acids, biogenic amines, and VOCs (Table S4). Lactic acid was the main organic acid, with an average concentration of 5.4 g/L, followed by acetic acid (0.8 g/L). Whereas glucose was absent, the lactose and galactose concentrations were both around 10 g/L in brine 1 but 7 and 12 times lower in brine 2, respectively. Twenty additional organic compounds had significantly different concentrations in both brines, of which benzaldehyde, benzyl alcohol, butyric acid, and hippuric acid were the most different, with concentrations at least three times higher in brine 1. For some organic compounds, brine sample 1A was odd compared to brine samples 1B and 1C. For instance, the concentrations of D-lactic acid were on average 937 mg/L in brine samples 1B and 1C but only 390 mg/L in brine sample 1A. The GABA concentrations were on average 222 mg/L in brine samples 1B and 1C but only 18 mg/L in brine sample 1A. Moreover, the galactose and lactose concentrations were 23% and 25% lower in brine sample 1A compared to brine samples 1B and 1C. In contrast, the acetic acid concentrations were 10% higher.

### Microbial counts of the brines

**Fig 9:**
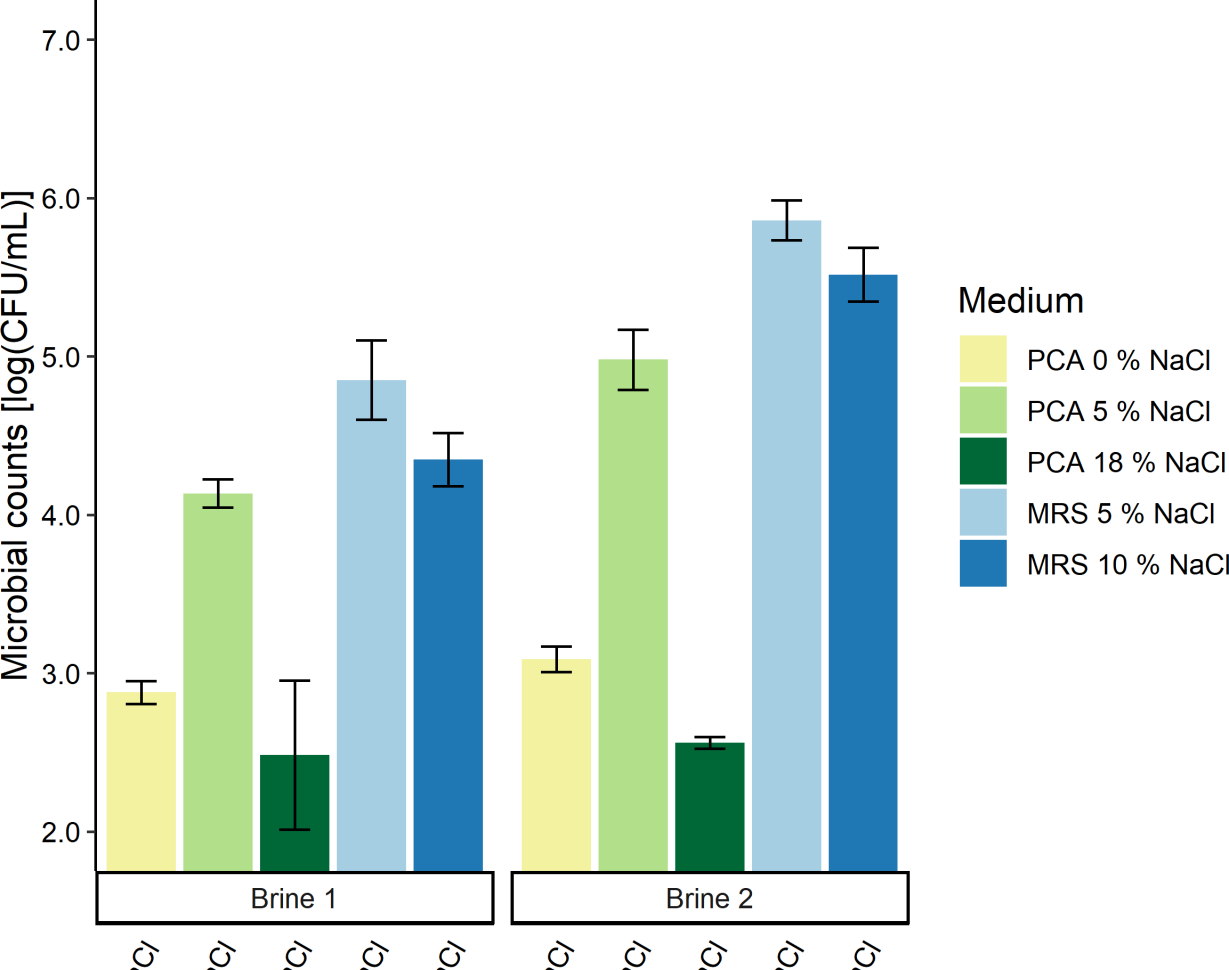
Microbial colony counts of brine 1 and brine 2 on different agar media; plate count agar (PCA) medium with 0%, 5%, and 18% (m/vol) NaCl (yellow, light green, and dark green, respectively) and de Man-Rogosa-Sharpe (MRS) agar medium with 5% and 10% (m/vol) NaCl (light blue and dark blue, respectively). Black flags indicate the standard deviations.

For the brine samples, the highest bacterial counts were found on MRS agar medium with 5% NaCl and were on average 4.8 and 5.9 log (CFU/mL) for brines 1 and 2, respectively (Fig. 9). The counts on PCA medium with 5% NaCl were almost one log lower compared to those on MRS agar medium with 5% NaCl, but they were significantly higher compared to the counts on PCA medium without and with 18% NaCl. The counts of brine 2 were significantly higher on PCA medium with 5% NaCl and MRS agar medium with 5% and 10% NaCl compared to those of brine 1.

Whereas all isolates of both MRS agar media examined were identified as *T. halophilus*, the different PCA media showed more diversity and harbored mainly yeasts (Fig. S1). *Debaryomyces hansenii* was the most abundant yeast species, followed by *Yamadazyma triangularis* that was only found on PCA medium with 18% NaCl.

### Microbial counts of the rennets

Since all 108 isolates of the 6 brine samples on MRS agar media with 5% and 10% NaCl were identified as *T. halophilus*, and, hence, not any *Loil. rennini* was isolated from the brine, despite its high relative abundance in the metagenetics data, it was assumed that *Loil. rennini* could not grow well on the agar media applied, although the type strain grew on MRS and salt-enriched MRS agar media. Hence, a liquid medium allowing the growth of *Loil. rennini* was needed to assess the growth of *Loil. rennini* from rennets. Of the 6 media tested, MRS medium with 33% (m/vol) brine (MRSb) showed the best growth for both strains.

After microbial enrichment in MRSb medium and plating on MRSb agar medium, 2 of the 7 commercial rennet samples indeed resulted in growth, whereas one sample showed only growth on MRSb agar medium for one of the triplicates (Table S5). The remaining 4 samples did not display growth, and 3 of these samples also led to low DNA concentrations from the cell pellets of the rennets. All isolates taken from the agar media with growth were identified as *T. halophilus*.

### Metagenetics of the brines and rennets

**Fig 10:**
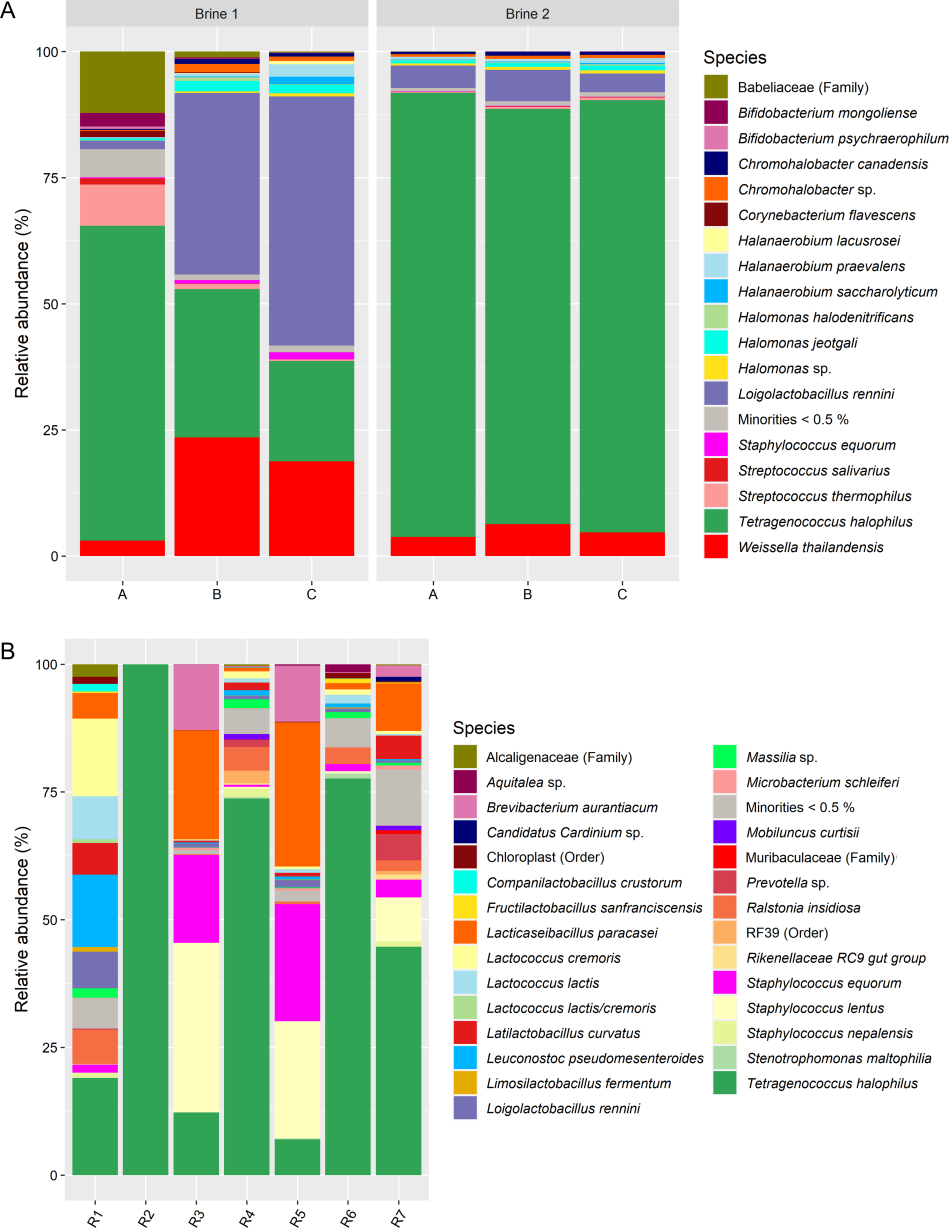
Taxonomic assessment of bacteria at species level, expressed as relative abundances based on amplicon sequence variants of the full-length 16S rRNA gene, for (A) brine 1 and brine 2 at the 3 different sampling locations in the brine baths (A, B, and C) and (B) seven commercial rennet samples (R1–R7).

The most abundant bacterial species in the brine was *T. halophilus*, followed by *Loil. rennini* and *W. thailandensis*, which were especially abundant in brine samples 1B and 1C (Fig. 10A). Additionally, *Chromohalobacter* spp., *Halanaerobium* spp., *Halomonas jeotgali*, *St. equorum*, *S. thermophilus*, and species of the Babeliaceae family were found at low relative abundances. The alpha diversity was 3.07 ± 0.63 and 1.37 ± 0.09 for brines 1 and 2, respectively, which were significantly different (*P*-value of 0.04). The *P*-value associated with the beta diversity between both brines was 0.10. Brine 1 had thus a higher diversity compared to brine 2 but did not display a significantly different microbial profile.

The seven commercial rennets sampled contained a broad variety of bacteria (Fig. 10B), with *T. halophilus*, *Lacc. paracasei*, *Staphylococcus lentus*, *St. equorum*, and *Brevibacterium aurantiacum* as the most prevalent species. There were no significant differences in alpha diversity and beta diversity regarding the two rennet suppliers, the ability to display growth on agar media after microbial enrichment, and the DNA concentrations of the rennet samples (Table S5).

The most abundant *Loil. rennini* amplicon sequence variants (ASVs) of the brine and rennet samples corresponded with the most abundant ASVs of the cheese samples (Fig. S2). Additionally, the brine and cheese samples had their most abundant *T. halophilus* ASVs in common. The ASV profiles of the rennets were similar but completely different from those of the brine and cheese samples.

### Impact of brine bacteria on the brine metabolites

#### Pearson correlations

Among the 6 different brine samples, the relative abundances of *Loil. rennini* and *W. thailandensis* were highly correlated (Pearson correlation), whereas these species were negatively correlated with the presence of *T. halophilus. Tetragenococcus halophilus* was significantly positively correlated with acetic acid, L-lactic acid, 2-phenylethylamine, and tryptamine and negatively correlated with the carbohydrates galactose and lactose, the SCFAs butyric acid, 3-methyl-butanoic acid, and hexanoic acid, the organic acids citric acid, D-lactic acid, hippuric acid, succinic acid, and uric acid, the amino acids GABA, glycine, histidine, isoleucine, leucine, serine, and threonine, the biogenic amine spermidine, and the VOCs 2,3-butanediol, 2,3-butanedione, and benzaldehyde (Table S6). The correlations between all forementioned organic compounds and *Loil. rennini* and *W. thailandensis* were the opposite of those between these compounds and *T. halophilus*. In particular, *Loil. rennini* had a positive correlation of 0.95 with GABA in the brine samples (and 0.76 in the cheese samples). In the cheeses, the relative abundance of *T. halophilus* was strongly associated with that of *Loil. rennini*, and the correlation between *T. halophilus* and GABA was moderately positive in the cheese samples (0.46) but strongly negative in the brine samples (−0.87). Additionally, the relative abundance of *S. thermophilus* was strongly correlated with that of members of the Babeliaceae family. The latter bacteria were significantly positively correlated with the concentrations of acetophenone, 2-phenyl ethanol, dimethyl sulfone, and phenyl acetaldehyde.

#### Biogenic amine production by brine microorganisms in simulation media

It was further shown that the brine indeed contained species and/or enzymes that led to biogenic amine formation (Fig. S3). After 1 week of fermentation using brine as inoculum, the concentrations of cadaverine, histamine, and putrescine were 23, 17, and 21 mg/L, respectively, in a medium optimal for biogenic amine production, and 10, 4, and 3 mg/L, respectively, in a cheese medium.

## DISCUSSION

Early and late gas production are well-known undesired traits during hard and semi-hard cheesemaking because of the resulting texture defects. However, even in the case of the use of high-quality milk and the application of hygienic processing conditions, unwanted gas production can occur, which results in cheeses with cracks. Although late blowing caused by *C. tyrobutyricum*, *P. freudenreichii* subsp. *shermanii*, and some species of *Lactobacillaceae* are usually under control, the underlying metabolic pathways leading to unexpected crack defects in long-ripened Gouda cheese were not known. The systematic metabolomic and microbiological analysis of the present study could unravel this.

The Gouda cheeses with cracks examined in the present study had a completely different metabolite profile compared to Gouda cheeses without cracks of similar ages. The key compound with higher concentrations in the Gouda cheeses with cracks was GABA, which is produced by the decarboxylation of glutamate, a major amino acid present in the cheese matrix. Per mole of glutamate converted, one mole of GABA and one mole of carbon dioxide are produced. Hence, an extensive glutamate decarboxylation led to excessive carbon dioxide formation (*in casu* 2,213 mg of glutamate produced per kilogram of cheese in the zones with cracks is equivalent with 21.46 mmol of carbon dioxide per kilogram of cheese). Although excessive carbon dioxide production is not desirable, the non-proteinogenic amino acid GABA is not a toxic compound. In contrast, it is an inhibitory neurotransmitter with health-promoting properties, such as lowering of the blood pressure and enhancing relaxation and immunity (29–31). GABA concentrations of 177–1,290 mg/kg have been reported for Gouda cheese (32, 33). Whereas the saturation concentration of carbon dioxide in the Gouda cheese matrix is about 36 mmol/kg cheese, eye formation starts already at 18 mmol/kg because of common carbon dioxide production (22). Since all cheeses with cracks had already eyes as a result of desired milk citrate conversion by the starter LAB, the carbon dioxide concentration was above 18 mmol/kg before the production of GABA took place. Hence, the GABA produced resulted in carbon dioxide concentrations exceeding the saturation concentration. Earlier, glutamate decarboxylation has been considered as a major contributor to eye formation in Gouda cheeses, provided that decarboxylation already happens in the early ripening stage, when the cheese texture is soft and elastic (34, 35). However, GABA concentrations in Gouda cheese are generally low, especially in the first half year of ripening (21). Moreover, as the Gouda cheese starter cultures applied did not contain any known glutamate decarboxylase activity, GABA was likely produced by the NSLAB at later ripening stages, when the cheese had a more firm texture, which resulted in cracks.

The NSLAB present in the Gouda cheeses examined were mainly *Lacc. paracasei* and *St. equorum*, as determined culture dependently, with no differences between the zones with cracks and the zones without cracks, hence, not being responsible for excessive carbon dioxide production. Whereas *Lacc. paracasei* is a common cheese NSLAB (16, 36), *St. equorum* is less prevalent but has been found in Gouda cheeses as well (37), *in casu* when plating on PCA medium (this study). However, there was probably a plating bias since both NSLAB species were only present at low relative abundances when a culture-independent amplicon-based high-throughput sequencing was applied. In contrast, the latter method revealed *Loil. rennini* and *T. halophilus* as the two most abundant NSLAB species in the zones with cracks of the Gouda cheeses examined. Based on the correlation analysis of these NSLAB and GABA production in the cheese and brine samples, *Loil. rennini* was most likely the GABA producer leading to cracks. Moreover, the latter species has been found in Gouda cheeses with phenolic off-flavors and cracks before (24), although it is also considered as a key species for the typical flavor of traditional Greek Kopanisti and Mana cheeses (38, 39). With respect to such a phenolic flavor, *Loil. rennini* positively correlated with the phenolic compounds benzaldehyde and benzyl alcohol present in the long-ripened cheeses. Since the Gouda cheeses with cracks had a completely different metabolite profile compared to similar Gouda cheeses without cracks and without abundant *Loil. rennini* (21), the latter species will probably contribute to a specific flavor in cheese that can be desired or undesired, depending on the strength of the flavor (in turn compound concentration dependent), the cheese type, and personal preferences.

Although various LAB species can produce GABA, strains of *Loil. rennini* are known for their high GABA production that is not hampered by 12% salt and 4% lactic acid in the medium (40). Generally, bacteria produce GABA to enhance their acid resistance and generate extra ATP (41). Hereto, a glutamate/GABA antiporter (GadC) takes up glutamate into the cell for decarboxylation to GABA by a glutamate decarboxylase (GadA or GadB), producing carbon dioxide and consuming an intracellular proton (42). GABA is then excreted by the antiporter, resulting into a less acidic and more negatively charged cytosol. The resulting proton motive force can then be used by the F_0_F_1_-ATPase to generate ATP. The MAG of *Loil. rennini* retrieved in the present study contained all necessary genes for GABA production with ATP generation, namely *gadC* and *gadB* and all eight subunits of the F_0_F_1_-ATPase system. In contrast, the *Lc. laudensis* MAG also contained the *gadB* gene but not the antiporter *gadC*, which is necessary for GABA production (43). Whereas GABA production by *T. halophilus* strains has been described before (44), the lack of correlation between *T. halophilus* and GABA production and the absence of the necessary genes in the *T. halophilus* MAG retrieved in the present study suggested that GABA production was a strain-dependent feature for this species.

Also, the production of biogenic amines contributes to an enhanced acid resistance. The MAG of *Loil. rennini* contained two different inducible ornithine decarboxylase genes and a putrescine/ornithine antiporter gene. A similar system has been described for *Ligl. saerimneri*, with two genes similar to ornithine decarboxylases, of which one is an ornithine decarboxylase that elaborates putrescine, and one is a lysine decarboxylase that elaborates cadaverine (45). The antiporter present in *Ligl. saerimneri* catalyzes both ornithine/putrescine and lysine/cadaverine exchange. Whereas one of the ornithine decarboxylases found in the MAG of *Loil. rennini* was similar to the *Ligl. saerimneri* lysine decarboxylase, the other ornithine decarboxylase was similar to the *Ligl. saerimneri* ornithine decarboxylase. Moreover, the antiporter of *Ligl. saerimneri* shared a high similarity with the putrescine/ornithine antiporter found in the MAG of *Loil. rennini*, suggesting that the latter had also an antiporter capable of both ornithine/putrescine and lysine/cadaverine exchange. This genetic potential corroborated with the positive correlation between *Loil. rennini* and both cadaverine and putrescine, and its negative correlations with lysine and ornithine. In the brines, *Loil. rennini* was only significantly correlated with putrescine since almost no lysine was present. Most likely, *Loil. rennini* was able to produce cadaverine and putrescine in a similar way as it produced GABA, resulting in extra carbon dioxide production, an intracellular pH increase, and extra ATP generation owing to a proton motive force. Moreover, the carbon dioxide produced from cadaverine and putrescine (10.08 and 4.66 mmol of carbon dioxide per kilogram of cheese, respectively) increased the crack formation. Besides the adverse health effect of cadaverine and putrescine (46), these compounds can also lead to fecal and putrid off-flavors (47, 48), which were indeed perceived in the Gouda cheeses with a crack defect. Finally, the MAG of *Loil. rennini* contained a tryptophane decarboxylase, capable of tryptamine production. This enzyme has also a weak activity on phenylalanine and tyrosine, leading to 2-phenylethylamine and tyramine, respectively (49). This could probably explain the significantly higher concentrations of these biogenic amines in the zones with cracks of the Gouda cheeses examined, although these concentrations were low compared to those of putrescine and cadaverine. A tryptophane/tryptamine antiporter was not found in the MAG of *Loil. rennini,* and hence, the lack of an efficient transporter might result in limited tryptamine production. Although the histamine concentrations were significantly higher in the zones with cracks compared to the other sampling areas, a histidine decarboxylase was not found in any MAG. However, histidine decarboxylases can be encoded on plasmids, as has been described for *T. halophilus* (50, 51), and plasmid-encoded genes are mostly not retrieved via MAGs (52). Therefore, all contigs were screened for histidine-related genes before binning, but only a histidine/histamine antiporter (HdcP) of *T. halophilus* was found. Possibly, most *T. halophilus* strains in the Gouda cheeses of the present study contained the *hdcP* gene, whereas only a minority, which depends on the sequencing depth, also harbored the actual decarboxylase genes (*hdc*A and *hdc*B). Alternatively, *T. halophilus* contained unknown histidine decarboxylases, or the tryptophane decarboxylase of *Loil. rennini* had some histidine decarboxylase activity. However, not any cheese, brine, and rennet samples that were plated resulted in *Loil. rennini* isolates, although the type strain grew on MRS and salt-enriched MRS agar media. A phenotypical confirmation of any decarboxylase activity by *Loil. rennini* was therefore impossible. Alternatively, two liquid media inoculated with brine resulted in the production of biogenic amines, demonstrating the decarboxylation abilities of one or more species present in the brine.

Finally, the higher concentrations of hexanal and nonanal in the Gouda cheeses with cracks could be ascribed to increased lipid oxidation. It is indeed known that high pressure treatments increase lipid oxidation in foods (53). Although the increased pressure in the cheeses with cracks cannot be considered as high, the long time of increased exposure to this increased pressure might still have resulted in a slightly increased lipid oxidation. Additionally, it might be related to the lipolytic properties that have been reported for *T. halophilus* (54).

Two possible inoculation sources of *Loil. rennini* and *T. halophilus* were identified, namely the brine and rennet used. Brines have already been shown to harbor *Loil. rennini* and *T. halophilus* (55) and have been identified as inoculation source of these NSLAB species in Gouda cheese rinds (21). The ASV profile of *T. halophilus* found in the Gouda cheeses of the present study was more similar to that of the brine than to that of the rennet, indicating that the brine was the most plausible inoculation source. Moreover, all cheeses had been brined in brine 1, which contained more *Loil. rennini* compared to brine 2. However, the brines and rennets were sampled more than 1 and 2 years after the production of these cheeses, respectively, which may have led to temporal variation of the ASVs. Especially the rennet, which is produced batchwise, might vary in ASV profile over time, whereas the brine resembles an ecosystem under continuous fermentation, as it was never replaced and, hence, is more likely to maintain the same strains and thus a similar ASV profile. Consequently, it could not be excluded that the contamination of the Gouda cheeses with both NSLAB species was caused by a batch of rennet with a higher bacterial load than usual, but more likely, the inoculation was caused by a more efficient carryover of bacteria from the brine toward the cheeses. This could be a result of less efficient pressing, resulting in small eyes in the rind before brining. The existence of small eyes in the rind could also explain why the Gouda cheeses with cracks had a higher salt-in-moisture content compared to those without cracks of similar age. Additionally, small channels that were formed from the rind toward the core could have led to local hotspots with higher salt concentrations in the cheese body. These higher salt-in-moisture concentrations may have favored the growth of the salt-tolerant *Loil. rennini* and *T. halophilus* over common cheese NSLAB species. Moreover, given their co-occurrence in the Gouda cheeses and brines, they might display synergetic growth. Indeed, both species have been found together in spontaneously fermented, salted soybean products (56, 57) and fermented fish sushi (58), despite the relatively rare detection of *Loil. rennini* in fermented foods. Nevertheless, no genetic evidence based on the MAGs retrieved could support synergetic growth, although the decarboxylation activities by *Loil. rennini* as an acid stress response might have enhanced the growth of *T. halophilus* too. Indeed, the latter species is quite acid sensitive, with an optimal pH for growth between 7.0 and 9.0 (59). However, the low pH of around 5.0 in the brine did not prevent *T. halophilus* to be the most abundant species.

The metabolites in the brines demonstrated an active metabolism of the brine microorganisms. For example, brine 2 contained 10 times lower concentrations of lactose and galactose and 10 times higher microbial counts on MRS agar medium compared to brine 1. Indeed, *T. halophilus* is capable of lactose and galactose utilization (59). A higher relative abundance of *T. halophilus* in brine sample 1A probably caused the lower lactose and galactose concentrations in this brine location compared to brine samples 1B and 1C. Additionally, the presence of *Loil. rennini* was positively correlated with higher concentrations of D-lactic acid and GABA, which corroborated with its genetic potential according to its MAG. Differences between both brines might be related to their age. Brine 1 was around 30 years old, whereas brine 2 was only 10 years old. The younger brine had thus a higher microbial load and likely a more active carbohydrate consumption by the microorganisms present. The salt concentration in the brines was kept constant by the dairy company using a brine density meter, but since the brines were never refreshed, a gradual increase of cheese compounds built up over the years. These compounds also contributed to the density of the brine, resulting in an overestimation of the salt concentrations measured. As a result, the real salt concentration of the brine gradually decreased, which might have led to a lower selection pressure than expected. This was reflected by a higher alpha diversity in brine 1 compared to brine 2, and in particular an increased relative abundance of *Loil. rennini* and *W. thailandensis*, which are less salt tolerant compared to *T. halophilus* (54, 59).

It has been suggested that *T. halophilus* in the brine originates from the sea salt used (55, 60). However, *T. halophilus* has not been detected in table salt or salterns up to now (61, 62). The present study demonstrated that *T. halophilus* was abundant in the cheese rennet. This rennet probably causes its inoculation in the brine through the brining of the cheeses and thus not the salt as previously assumed (55), but the salt does allow the selection of halotolerant bacteria. *Tetragenococcus halophilus* has been found in the rumen of green roughage-fed buffalo (63) and occurs as a minor species in various raw milk cheeses (50, 54, 64–66), suggesting an animal origin. Similarly, *Loil. rennini* has been found in silages (67) and the related species *Loigolactobacillus coryniformis* in cow rumen (68). In general, the most prevalent microorganisms in animal rennets are LAB species, with *Lactiplantibacillus plantarum* and *Levilactobacillus brevis* as the main species determined in a culture-dependent way (69) and *Lactobacillus crispatus*, *Limosilactobacillus reuteri*, and *S. thermophilus* as the most abundant ones when determined in a culture-independent way (70). The latter study has also detected *Tetragenococcus* and several unidentified *Lactobacillaceae*, including *Loigolactobacillus* species. It seems that the microbiology of rennets is highly diverse and differs greatly between producers. Its microbiota can, however, affect the cheese quality either positively or negatively. Furthermore, the rennet microbiota can influence the cheese microbiota not only directly but also indirectly via the brine as an intermediate inoculation source, which under extreme situations might allow a single batch of rennet to influence the cheese household microbiota for years.

### Conclusion

*Loigolactobacillus rennini*, an uncommonly detected NSLAB species in Gouda cheese, was associated with high concentrations of GABA, cadaverine, and putrescine in cheeses with crack defects. Its metagenome-assembled genome contained all the necessary genes to produce these compounds, resulting in excessive carbon dioxide formation and cracks in the cheese body, as well as unpleasant off-flavors due to the biogenic amines produced. Although *Loil. rennini* was strongly associated with another NSLAB species, *T. halophilus*, there was no evidence that the latter contributed to the crack formation. In general, the Gouda cheeses with a crack defect had a significantly different metabolite profile compared to the cheeses without cracks, suggesting that, alternatively, *Loil. rennini* and/or *T. halophilus* could be used as adjunct starters to steer the cheese flavor to another direction favoring diversification. Whereas high concentrations of GABA could be desired because of its health-promoting properties, a thorough selection of such functional starter culture strains should be done to ensure that no biogenic amines are produced. Both NSLAB species were abundant in the brine, the most plausible inoculation source of the Gouda cheeses, whereas *T. halophilus* was especially abundant in the rennet. The two brines used in the dairy company differed in age, which was reflected in a lower microbial load, higher carbohydrate concentrations, and a higher bacterial species diversity in the oldest brine. The bacterial diversity of the rennet was high, pointing it as another plausible NSLAB source.

## MATERIALS AND METHODS

### Samplings

#### Cheese selection and sampling

Gouda cheese wheels of 6 different batch productions, made in a European dairy factory, that displayed cracks and had an unpleasant, putrid off-flavor, were investigated. The age of the cheeses was 31 weeks. The cheeses were produced between August and October 2019. The cracks were scored as medium (M, two batches, referred to as M1 and M2), large (L, three batches, referred to as L1, L2, and L3), or extra large (XL, one batch), with a number (1, 2, or 3) (Fig. 1). Three cheese wheels were selected from batches M1 (referred to as M1a, M1b, and M1c) and L1 (referred to as L1a, L1b, and L1c) to investigate within-batch differences. This resulted in a total of 10 cheese wheels.

For sampling, each cheese wheel was cut into two halves, and a slice of 3 cm from the border of one halve was taken, vacuum packed, and stored at 4°C in the factory before transport to and further sampling in the research group’s laboratory. There, three areas of the cheese slice situated around the zones with cracks were excised, cut into pieces, and pooled to give one crack-zones sample per cheese wheel. Similarly, three areas of the cheese slice without cracks were excised, cut into pieces, and pooled to give one zones-without-cracks sample per cheese wheel. The cheese with crack score XL had too much cracks to allow a zones-without-cracks sample. In contrast, the cracks of cheese wheel M2 were not visible upon sampling in the laboratory, which therefore resulted in a sample of the core and the rind.

Finally, data of the cores and rinds of similar Gouda cheeses without cracks, made in the same dairy factory and ripened for 31 and 36 weeks, were considered for comparison purposes (21).

#### Brine and rennet sampling

As two brine baths were present in the factory (although the 10 Gouda cheese wheels examined were brined in brine 1), samples (1,000 mL) of these two brines (referred to as brine 1 and brine 2) were taken at three different locations of the baths (A, B, and C) in February 2021. Therefore, γ-irradiated jumbo pipettes (900 mm, high-density polyethylene; VWR International, Darmstadt, Germany) were used. Also, as animal rennet from two different commercial suppliers (A and B) was used for the Gouda cheese productions, seven different batches of animal rennets (referred to as R1–R7) were sampled (400 mL) between September 2021 and January 2022.

### Physicochemical parameter characterization of the Gouda cheese and brine samples

The physicochemical parameters pH, moisture content, and salt-in-moisture content of the Gouda cheese samples were determined, as described previously (21). The physicochemical parameters of similar Gouda cheeses without cracks, made in the same dairy factory, and ripened for 31 and 36 weeks, that have been reported previously (21), were used for comparison purposes. Also, the pH, temperature, and salt content of the brines were determined.

### Metabolomic analysis of the Gouda cheese and brine samples

A metabolomic analysis was performed for the Gouda cheese and brine samples, as described previously (21). Amino acids, biogenic amines, and other organic acids were quantified by ultra-performance liquid chromatography coupled to tandem mass spectrometry with an Acquity system equipped with an HSS T3 column and a TQ tandem mass spectrometer (Waters, Milford, Massachusetts, USA). In the case of D-lactic acid and L-lactic acid, an Astec Chirobiotic column (Supelco; Merck, Darmstadt, Germany) was used. The concentrations of short-chain fatty acids and volatile organic compounds were measured using ethyl acetate extracts by means of gas chromatography coupled to tandem mass spectrometry with a Trace 1300 gas chromatograph equipped with a TriPlus RSH autosampler, a DB-WAXetr column, and a TSQ 8000 EVO triple-quadrupole mass spectrometer (Thermo Fisher Scientific, Waltham, Massachusetts, USA). However, for the brine samples, the extracts for the determination of the SCFAs and VOCs targeted were made by adding 1.0 mL of brine sample to 9.0 mL of acetone (Sigma-Aldrich, St. Louis, Missouri, USA) supplemented with 100 µg/L of toluene-D8 (Sigma-Aldrich) as internal standard (IS). In the case of the brine samples, carbohydrates were measured as well, by high-performance anion exchange chromatography with pulsed amperometric detection using an ICS-5000 chromatograph (Dionex, Sunnyvale, California, USA) equipped with a CarboPac PA-20 column (Dionex), as described previously (71). Finally, the metabolite concentrations of similar Gouda cheeses without cracks, made in the same dairy factory and ripened for 31 and 36 weeks, that have been reported previously (21) were used for comparison purposes.

### Microbial colony enumeration and identification

#### Gouda cheese samples

Ten grams of Gouda cheese sample were resuspended with 90 mL of sterile saline (8.5 g/L of NaCl; Merck) in a stomacher (Laboratory Blender Stomacher 400; Seward, Worthing, West Sussex, UK) at medium speed for 5 min. Then, serial dilutions of these cheese suspensions were made with sterile saline. Each dilution (100 µL) was plated on plate count agar medium, containing 5.0 g/L of tryptone (Oxoid, Basingstoke, Hampshire, UK), 2.5 g/L of yeast extract (Oxoid), and 1.0 g/L of glucose (Merck), and de Man-Rogosa-Sharpe agar medium (Oxoid) supplemented with cycloheximide (200 mg/L, Merck) to enumerate total microorganisms and LAB, respectively. Plates were incubated at 30°C for 3 days.

After enumeration, agar media with the appropriate dilutions were washed with 1 mL of sorbitol buffer [1.2 M sorbitol (VWR International), 50 mM Tris-base (Calbiochem, San Diego, California, USA), pH 7.5], and the resulting cell suspensions were pelleted by microcentrifugation at 6,000 × *g* for 10 min. The cell pellets were subjected to a DNA extraction, as described previously (21). In the case of the cell suspensions from the MRS agar media, the first enzymatic step to lyse yeast cells was not performed. The DNA was used to amplify the bacterial full-length 16S rRNA gene, and the amplicons obtained were sequenced using PacBio technology. The sequence reads obtained were processed using the DADA2 pipeline, resulting in ASVs for species identification, as described previously (21).

#### Brine samples

The brine samples, serially diluted with sterile saline, were plated (100 µL) on PCA medium with 0%, 5%, and 18% (m/vol) NaCl and on MRS agar medium with 5% and 10% (m/vol) NaCl. Plates were incubated at room temperature for 7 days in the case of PCA medium and for 10 days and anaerobically (AnaeroGen; Thermo Fisher Scientific) in the case of MRS agar medium. After colony enumeration, 9 colonies [8 and 6 in the case of PCA medium with 0% and 18% (m/vol) NaCl, respectively] were randomly picked from the agar media and transferred to liquid MRS and tryptone-yeast extract [5.0 g/L of tryptone (Oxoid), 2.5 g/L of yeast extract (Oxoid), and 1.0 g/L of glucose (Merck)] media, supplemented with the appropriate NaCl concentration. After 3 days of incubation at room temperature, cell pellets were obtained by microcentrifugation at 6,000 × *g* for 10 min, which were washed in a Tris-ethylene diaminetetraacetic acid (EDTA)-sucrose buffer [50 mM Tris base (Calbiochem), 1 mM EDTA (Sigma-Aldrich), and 6.7% (m/vol) sucrose (Merck); pH 8.0]. These pellets were subjected to genomic DNA extraction with a NucleoSpin 96 Tissue Kit (Macherey Nagel, Düren, Germany), according to the manufacturer’s instructions. For dereplication of the isolates, (GTG)_5_-PCR fingerprinting of genomic DNA was performed, as described previously (72). The dereplication and numerical clustering were conducted with BioNumerics 5.1 software (Applied Maths, Sint-Martens-Latem, Belgium), and the species identity of representative isolates of each cluster was revealed by 16S rRNA gene Sanger sequencing, as described previously (72).

#### Rennet samples

In the case of the rennet samples, isolation of *Loil. rennini* would be the main purpose of plating, as it was thought to be the main species of interest in the rennets. Since not any *Loil. rennini* isolate from the cheese or brine samples was found on MRS or salt-enriched MRS agar media, liquid MRS medium and 5 adapted media were used to assess the growth of two *Loil. rennini* strains, namely *Loil. rennini* LMG 23902^T^ and *Loil. rennini* LMG 23903. These adapted media were MRS medium with 5 g/L of xylose; cheese medium (70), made with Gouda cheese (without cracks) comparable to the cheeses under investigation; MRS medium with 33% (m/vol) cheese medium; a medium with 50% (m/vol) water, 50% (m/vol) brine from the dairy company, and 5 g/L of xylose; and MRS medium with 33% (m/vol) brine (MRSb). The use of xylose in some of the media reflects the capability of *Loil. rennini* to use it as an energy source, which is not possible by *T. halophilus* and, hence, selects for the growth of *Loil. rennini* (25, 59, 73).

Finally, samples of the rennets were plated after microbial enrichment. Hereto, 1 mL of rennet was mixed with 5 mL of MRSb medium, incubated for 7 days at room temperature, and plated on MRSb agar medium that was incubated anaerobically for 7 days at room temperature. This was done in triplicate, after which a binary growth/no growth score was given. Colonies were randomly picked from the agar medium and transferred to liquid MRSb medium, grown at room temperature for 2 days, and the cell pellets obtained were further treated for DNA extraction and species identification, as described above for the brine isolates.

### Cell pelleting, DNA extraction, and metagenetics of the Gouda cheese, brine, and rennet samples

A metagenetic analysis was performed on the Gouda cheese, brine, and rennet samples. Cell pelleting of the cheese samples was performed as described previously (21). Briefly, cheese samples were mixed with a sterile solution of 2% (m/vol) sodium citrate (Sigma-Aldrich) in a stomacher (Seward), and the resulting suspension was centrifuged at 4,500 × *g* for 20 min, followed by a resuspension in sorbitol buffer and another centrifugation at 6,000 × *g* for 10 min. The same procedure was used for the brine and rennet samples, except that those samples were centrifuged as such (without mixing with sodium citrate). DNA extraction, full-length 16S rRNA amplicon-based high-throughput sequencing using PacBio technology, and data processing using the DADA2 pipeline, resulting in ASVs for species identification, were performed as described previously (21).

### Metagenomic analysis of the Gouda cheese samples

A metagenomic analysis was performed on the 10 crack-zones samples and the 9 zones-without-cracks samples of the Gouda cheeses examined. Therefore, Illumina NovaSeq sequencing was performed, as described previously (74). Further processing of the metagenomic sequence reads was also performed as described previously (71). Co-assembly of the metagenomes was done with MEGAHIT (75), the assembly was imported in anvi’o (76), mapping was done using Bowtie 2 (77), and contig binning was done with CONCOCT (78). Bins were manually refined, based on stable G + C content and consistent read coverage, to obtain bins with a high completion (>75%) and low redundancy (<10%). The resulting bins are further referred to as metagenome-assembled genomes. Finally, annotation was carried out with PROKKA [version 1.14.6; (79)]. Based on the MAGs, annotated inducible ornithine decarboxylases and transporters were aligned by the BLAST algorithm blastp toward the following proteins of *Ligl. saerimneri* 30a: inducible ornithine decarboxylase [OdcI, National Center of Biotechnology Information (NCBI, Bethesda, Maryland, USA) Protein accession number WP_235806344.1], lysine decarboxylase (Ldc, Protein accession number WP_009553967.1), and a putrescine transporter (PotE, Protein accession number WP_009553966.1). The BLAST algorithm tblastn was used to align all contigs before binning toward the histidine decarboxylases of *Ligl. saerimneri* 30a (HdcA, UniProt accession P00862; HdcB, UniProt accession P09111), the histidine/histamine antiporter of *Ligl. saerimneri* 30a (HdcP, UniProt accession B3RFM4), and the homologous proteins of *T. halophilus* (HdcA, UniProt accession B1B5I3; HdcB, UniProt accession B1B5I4; HdcP, UniProt accession B1B5I2). For the MAGs of *Loil. rennini* and *T. halophilus*, KEGG pathways were retrieved via anvi’o (76), and the shared and unique KEGG functions of these MAGs were visualized using https://www.genome.jp/kegg/mapper/color.html (80) to detect possible synergies between both species.

### Biogenic amine production by brine microorganisms in simulation media

To investigate if the brine microbiota could lead to biogenic amine production, small-scale fermentation processes with an inoculum of 1% (vol/vol) brine 1 (brine sample B) were performed in sterile, closed 15 mL Falcon tubes (Sarstedt, Nümbrecht, Germany) at 15°C. Therefore, two different media were used. A liquid medium optimal for the production of biogenic amines was prepared as defined before (81) but without meat extract and supplemented with 5 g/L of NaCl. Also, a cheese medium as described above was used. Samples were taken after 0, 1, 2, and 4 weeks of fermentation, and biogenic amines were quantified as described previously (21).

### Statistical analysis

All statistical analyses were performed in R (www.R-project.org; version 4.1.0). A scaled principal component analysis was performed to compare the concentrations of all metabolites between the crack-zones samples, the zones-without-cracks samples, and core and rind samples of similar Gouda cheeses without crack problems from a previous study (21). A Wilcoxon rank sum test was performed to compare differences in physicochemical parameters and metabolite concentrations between the crack-zones samples, the zones-without-cracks samples, and the Gouda cheese samples from the previous study. Pearson correlation coefficients were calculated between metabolites and species. Only species with relative abundance above 5% were considered. An unpaired two-sample *t*-test for unequal variances (Welch’s *t*-test) was performed to compare differences between the metabolite concentrations in the two brines. Microbial intra-sample diversity (alpha diversity) was assessed by calculating the inverse Simpson diversity index, applying the vegan package (82). Microbial inter-sample diversity (beta diversity) was assessed by conducting a permutational multivariate analysis of variance, based on Bray-Curtis dissimilarity scores, applying the RVAideMemoire package (83). For all statistical tests, results with a *P*-value <0.05 were considered significantly different.

## Data Availability

The sequence data obtained are available under BioProject PRJEB64331. The PacBio amplicon sequences for the full-length 16S rRNA gene are available under accession numbers ERR11714512-ERR11714530, and the Illumina NovaSeq shotgun metagenomics sequences are available under accession numbers ERR11718190-ERR11718208 in the European Nucleotide Archive of the European Bioinformatics Institute (ENA/EBI).

## SUPPLEMENTAL MATERIAL

Supplemental tables and figures aem.01655-23-s0001.docx Tables S1 to S6 and Fig. S1 to S3.

ASM does not own the copyrights to Supplemental Material that may be linked to, or accessed through, an article. The authors have granted ASM a non-exclusive, world-wide license to publish the Supplemental Material files. Please contact the corresponding author directly for reuse.
