# Supplementary material for: Decarboxylase activity of the non-starter lactic acid bacterium Loigolactobacillus rennini gives crack defects in Gouda cheese through the production of γ-aminobutyric acid
Source: Appl Environ Microbiol. 2024 Jan 17;90(2):e01655-23. doi: 10.1128/aem.01655-23 (PMC10880667; doi:10.1128/aem.01655-23)
Supplement: Supplemental tables and figures — Tables S1 to S6 and Fig. S1 to S3. [file aem.01655-23-s0001.docx]

Supplementary Material

Decarboxylase activity of the non-starter lactic acid bacterium *Loigolactobacillus rennini* gives crack defects in Gouda cheese through the production of γ-aminobutyric acid

Hannes Decadt, Louise Vermote, Cristian Díaz-Muñoz, Stefan Weckx, Luc De Vuyst #

# **Correspondence:** Prof. Dr. ir. Luc De Vuyst: luc.de.vuyst@vub.be

# 1 Supplemental tables

**Table S1**. Pearson correlation between organic compounds and physicochemical parameters of Gouda cheeses with an age of 31 weeks and a crack defect and lactic acid bacterial species found in these cheeses with relative abundance above 5 %. Significant correlations are indicated in bold.

| Gouda cheese characteristic | *Lactococcus cremoris* | | *Lactococcus lactis* | | *Leuconostoc  pseudo-mesenteroides* | | *Loigolacto-bacillus*  *rennini* | | *Tetragenococcus halophilus* | |
| --- | --- | --- | --- | --- | --- | --- | --- | --- | --- | --- |
| *Organic compounds* |  | |  | |  | |  | |  | |
| Acetic acid | 0.35 | 0.13 | | -0.08 | | -0.17 | | -0.44 | |  |
| Acetoin | **0.54** | **0.57** | | -0.22 | | **-0.61** | | -0.42 | |  |
| Acetophenone | 0.23 | 0.07 | | 0.44 | | -0.26 | | -0.34 | |  |
| Alanine | -0.27 | -0.05 | | -0.33 | | 0.29 | | 0.20 | |  |
| Arginine | -0.06 | -0.42 | | -0.31 | | 0.44 | | 0.21 | |  |
| Asparagine | -0.02 | 0.19 | | -0.04 | | 0.00 | | -0.29 | |  |
| Aspartic acid | **-0.46** | -0.31 | | -0.25 | | **0.54** | | 0.39 | |  |
| Benzaldehyde | **-0.69** | **-0.51** | | -0.44 | | **0.80** | | **0.76** | |  |
| Benzyl alcohol | **-0.52** | **-0.54** | | -0.13 | | **0.71** | | **0.50** | |  |
| 2,3-Butanediol | 0.33 | 0.37 | | **0.70** | | **-0.56** | | **-0.66** | |  |
| 2,3-Butanedione | 0.16 | 0.18 | | **-0.66** | | -0.05 | | 0.15 | |  |
| Butyric acid | 0.05 | -0.24 | | -0.34 | | 0.30 | | -0.05 | |  |
| Cadaverine | **-0.50** | -0.32 | | **-0.49** | | **0.66** | | 0.45 | |  |
| Citric acid | -0.27 | -0.36 | | 0.11 | | 0.42 | | 0.12 | |  |
| Citrulline | **0.71** | 0.40 | | -0.04 | | **-0.63** | | **-0.59** | |  |
| δ-Decalactone | 0.07 | -0.08 | | 0.01 | | -0.08 | | 0.20 | |  |
| δ-Dodecalactone | 0.02 | -0.24 | | -0.06 | | 0.08 | | 0.32 | |  |
| Dimethyl sulfone | 0.27 | 0.15 | | 0.39 | | -0.32 | | -0.42 | |  |
| D-Lactic acid (% of  total lactic acid) | -0.29 | 0.45 | | -0.07 | | -0.04 | | -0.16 | |  |
| Fumaric acid | 0.12 | 0.05 | | **0.53** | | -0.29 | | -0.22 | |  |
| γ-Aminobutyric acid | -0.44 | **-0.57** | | -0.21 | | **0.71** | | **0.46** | |  |
| Gluconic acid | 0.26 | 0.08 | | -0.04 | | -0.14 | | -0.27 | |  |
| Glucuronic acid | 0.30 | **0.57** | | 0.03 | | **-0.58** | | -0.32 | |  |
| Glutamic acid | 0.21 | **0.50** | | -0.03 | | -0.44 | | -0.34 | |  |
| Glutamine | -0.08 | **0.49** | | 0.02 | | -0.24 | | -0.20 | |  |
| Glycine | -0.11 | -0.03 | | -0.30 | | 0.21 | | 0.01 | |  |
| Hexanal | **-0.63** | -0.41 | | **-0.51** | | **0.73** | | **0.72** | |  |
| Hexanoic acid | -0.01 | **-0.49** | | **-0.55** | | **0.47** | | 0.30 | |  |
| Hippuric acid | -0.14 | -0.18 | | 0.02 | | 0.22 | | 0.10 | |  |
| Histamine | -0.29 | -0.36 | | -0.15 | | 0.35 | | **0.54** | |  |
| Histidine | **-0.46** | -0.20 | | -0.35 | | **0.49** | | 0.42 | |  |
| Isobutyric acid | -0.36 | **-0.63** | | -0.30 | | **0.77** | | 0.38 | |  |
| Isoleucine | -0.24 | -0.10 | | -0.11 | | 0.26 | | 0.09 | |  |
| Lactic acid | 0.37 | 0.19 | | 0.35 | | -0.33 | | **-0.58** | |  |
| Leucine | -0.09 | 0.04 | | -0.23 | | 0.17 | | -0.07 | |  |
| Lysine | **0.64** | **0.63** | | 0.43 | | **-0.88** | | **-0.77** | |  |
| Maleic acid | -0.14 | 0.40 | | 0.02 | | -0.17 | | -0.18 | |  |
| Malic acid | -0.25 | -0.29 | | **-0.51** | | **0.48** | | 0.36 | |  |
| Malonic acid | -0.27 | -0.43 | | -0.32 | | **0.50** | | 0.39 | |  |
| Methional | 0.35 | 0.28 | | **-0.52** | | -0.23 | | -0.17 | |  |
| Methionine | -0.19 | -0.07 | | -0.13 | | 0.23 | | 0.03 | |  |
| 2-Methyl-butanoic acid | **-0.48** | **-0.61** | | -0.37 | | **0.83** | | **0.46** | |  |
| 3-Methyl-butanoic acid | **-0.47** | **-0.59** | | -0.37 | | **0.83** | | 0.44 | |  |
| Nonanal | -0.23 | -0.05 | | -0.31 | | 0.27 | | 0.14 | |  |
| 2-Nonanone | -0.28 | -0.23 | | **-0.75** | | **0.53** | | 0.42 | |  |
| Octanoic acid | 0.37 | -0.25 | | -0.24 | | -0.10 | | 0.20 | |  |
| Ornithine | **0.60** | **0.62** | | **0.62** | | **-0.92** | | **-0.78** | |  |
| Orotic acid | **-0.54** | -0.09 | | **-0.51** | | 0.45 | | **0.60** | |  |
| Oxalic Acid | 0.04 | -0.02 | | -0.03 | | -0.03 | | 0.00 | |  |
| 2-Pentanone | 0.10 | -0.35 | | -0.25 | | 0.19 | | 0.16 | |  |
| Phenol | **-0.53** | -0.31 | | 0.33 | | 0.36 | | 0.43 | |  |
| Phenyl acetaldehyde | **0.59** | 0.31 | | -0.16 | | **-0.48** | | **-0.48** | |  |
| Phenylalanine | -0.21 | 0.03 | | -0.26 | | 0.19 | | 0.13 | |  |
| 2-Phenylethyl amine | **-0.62** | **-0.53** | | **-0.48** | | **0.78** | | **0.79** | |  |
| Proline | -0.36 | -0.13 | | **-0.48** | | 0.43 | | 0.33 | |  |
| Propionic acid | **-0.58** | -0.41 | | -0.35 | | **0.71** | | **0.53** | |  |
| Putrescine | **-0.58** | -0.35 | | **-0.50** | | **0.73** | | **0.51** | |  |
| Pyruvic acid | -0.01 | -0.34 | | -0.36 | | 0.32 | | 0.23 | |  |
| Serine | **0.70** | **0.54** | | 0.43 | | **-0.88** | | **-0.73** | |  |
| Spermidine | 0.06 | 0.26 | | **0.56** | | -0.26 | | -0.45 | |  |
| Spermine | 0.24 | 0.34 | | **0.75** | | **-0.49** | | **-0.63** | |  |
| Succinic acid | -0.25 | -0.32 | | -0.37 | | **0.47** | | 0.31 | |  |
| Tetramethylpyrazine | **-0.61** | -0.37 | | -0.45 | | **0.69** | | **0.62** | |  |
| Threonine | **0.48** | **0.57** | | 0.27 | | **-0.69** | | **-0.66** | |  |
| Trimethylpyrazine | **-0.69** | **-0.51** | | -0.45 | | **0.80** | | **0.78** | |  |
| Tryptamine | **-0.67** | **-0.53** | | -0.32 | | **0.75** | | **0.82** | |  |
| Tryptophane | 0.15 | 0.27 | | -0.19 | | -0.20 | | -0.16 | |  |
| Tyramine | -0.31 | -0.35 | | -0.01 | | 0.40 | | 0.40 | |  |
| Tyrosine | 0.16 | 0.14 | | -0.09 | | -0.14 | | -0.20 | |  |
| Valeric acid | **0.47** | 0.20 | | **0.55** | | **-0.56** | | **-0.54** | |  |
| Valine | -0.19 | -0.02 | | -0.24 | | 0.23 | | 0.04 | |  |
| *Physicochemical parameters* | |  | |  | |  | |  | |  |
| Dry mass | **-0.46** | **-0.54** | | -0.24 | | **0.61** | | **0.69** | |  |
| pH | -0.30 | -0.14 | | -0.16 | | 0.36 | | 0.14 | |  |
| Salt-in-moisture | **-0.58** | **-0.46** | | -0.11 | | **0.72** | | 0.38 | |  |

**Table S2**. Statistics of the metagenome-assembled genomes (MAGs) retrieved from whole-community DNA isolated from the zones with cracks and the zones without cracks of Gouda cheeses with an age of 31 weeks and a crack defect.

| Species | Total length (nt) | Number of contigs | | N50 | | GC content (%) | | Completion (%) | | Redundancy (%) | | Number of Predicted genes by PROKKA |
| --- | --- | --- | --- | --- | --- | --- | --- | --- | --- | --- | --- | --- |
| *Lactocaseibacillus paracasei* | 2,319,253 | 187 | 18,585 | | 46.6 | | 97.2 | | 1.4 | | 2,185 | |
| *Lactococcus laudensis* | 1,921,923 | 135 | 23,530 | | 39.1 | | 93.0 | | 4.2 | | 1,874 | |
| *Loigolactobacillus rennini* | 2,097,550 | 136 | 24,965 | | 40.4 | | 98.6 | | 0.0 | | 2,037 | |
| *Tetragenococcus halophilus* | 2,397,461 | 161 | 23,669 | | 35.9 | | 98.6 | | 4.2 | | 2,371 | |
| *Weissella thailandensis* | 1,628,349 | 204 | 9,956 | | 38.9 | | 88.7 | | 0.0 | | 1,630 | |

**Table S3**. Physicochemical parameters of the different samples from two brines (1 and 2) and three locations in the brine baths (A, B, and C).

| Brine sample | pH | Temperature (°C) | | NaCl (g/L) |
| --- | --- | --- | --- | --- |
| 1A | 5.06 | 13.0 | 259 | |
| 1B | 4.98 | 12.9 | 187 | |
| 1C | 5.11 | 12.9 | 203 | |
| 2A | 4.94 | 13.6 | 232 | |
| 2B | 4.93 | 13.8 | 191 | |
| 2C | 4.84 | 17.4 | 208 | |

**Table S4**. Organic compounds present in two brines (1 and 2). Significant differences between the brines are indicated with * (*p* < 0.05), ** (*p* < 0.01), or *** (*p* < 0.001).

| Compound (mg/L) | Brine 1 | | | Brine 2 | |  |
| --- | --- | --- | --- | --- | --- | --- |
| *Carbohydrates* |  |  | |  |  |  |
| Galactose | 9183.50 | ± 1410.74 | 752.29 | | ± 3.42 | ** |
| Glucose | 0.00 | ± 0.00 | 0.00 | | ± 0.00 |  |
| Lactose | 10764.85 | ± 1830.26 | 1563.83 | | ± 44.02 | * |
| *Organic acids* |  |  |  | |  |  |
| Citric acid | 122.53 | ± 2.52 | 52.08 | | ± 3.04 | *** |
| Hippuric acid | 0.45 | ± 0.02 | 0.12 | | ± 0.03 | *** |
| D-Lactic acid | 755.14 | ± 315.93 | 430.06 | | ± 19.62 |  |
| L-Lactic acid | 4377.26 | ± 342.71 | 5209.31 | | ± 179.65 | * |
| Malic acid | 0.55 | ± 0.12 | 0.54 | | ± 0.05 |  |
| Succinic acid | 159.61 | ± 2.99 | 108.07 | | ± 2.34 | *** |
| Uric acid | 2.83 | ± 0.07 | 1.85 | | ± 0.10 | *** |
| *Short-chain fatty acids* |  |  |  | |  |  |
| Acetic acid | 816.99 | ± 44.09 | 858.44 | | ± 21.39 |  |
| Butyric acid | 67.46 | ± 2.13 | 22.36 | | ± 2.98 | *** |
| Hexanoic acid | 4.91 | ± 0.29 | 2.17 | | ± 0.30 | *** |
| Isobutyric acid | 3.08 | ± 0.09 | 2.36 | | ± 0.10 | *** |
| 3-Methyl-butanoic acid | 3.88 | ± 0.08 | 2.70 | | ± 0.05 | *** |
| Propionic acid | 17.56 | ± 1.14 | 20.58 | | ± 1.31 | * |
| Valeric acid | 0.84 | ± 0.09 | 0.81 | | ± 0.09 |  |
| *Amino acids* |  |  |  | |  |  |
| Alanine | 20.05 | ± 20.75 | 10.52 | | ± 10.26 |  |
| Arginine | 10.62 | ± 11.27 | 3.25 | | ± 3.84 |  |
| Asparagine | 4.72 | ± 3.80 | 3.71 | | ± 2.31 |  |
| Aspartic acid | 61.18 | ± 46.69 | 45.49 | | ± 59.64 |  |
| Citrulline | 2.37 | ± 1.91 | 3.12 | | ± 1.82 |  |
| Cysteine | 6.21 | ± 3.41 | 4.80 | | ± 4.71 |  |
| γ-Aminobutyric acid (GABA) | 153.58 | ± 117.91 | 58.17 | | ± 39.42 |  |
| Glutamic acid | 39.69 | ± 36.26 | 12.33 | | ± 10.79 |  |
| Glutamine | 5.32 | ± 6.40 | 1.30 | | ± 2.25 |  |
| Glycine | 29.97 | ± 20.86 | 11.77 | | ± 11.43 |  |
| Histidine | 5.92 | ± 3.57 | 1.35 | | ± 0.27 |  |
| Isoleucine | 37.20 | ± 33.31 | 3.10 | | ± 2.71 |  |
| Leucine | 150.27 | ± 124.88 | 35.40 | | ± 36.22 |  |
| Lysine | 0.07 | ± 0.12 | 0.00 | | ± 0.00 |  |
| Methionine | 9.72 | ± 6.85 | 4.31 | | ± 3.22 |  |
| Ornithine | 0.59 | ± 0.18 | 0.46 | | ± 0.46 |  |
| Phenylalanine | 22.69 | ± 17.24 | 6.75 | | ± 2.99 |  |
| Proline | 88.17 | ± 78.81 | 44.62 | | ± 32.03 |  |
| Serine | 29.89 | ± 25.91 | 3.17 | | ± 5.13 |  |
| Threonine | 42.34 | ± 37.60 | 6.83 | | ± 5.39 |  |
| Tryptophane | 0.00 | ± 0.00 | 0.00 | | ± 0.00 |  |
| Tyrosine | 0.78 | ± 0.80 | 1.42 | | ± 1.55 |  |
| Valine | 80.80 | ± 77.00 | 17.91 | | ± 20.26 |  |
| *Biogenic amines* |  |  |  | |  |  |
| Cadaverine | 62.13 | ± 1.84 | 62.19 | | ± 1.48 |  |
| Histamine | 29.90 | ± 2.16 | 32.06 | | ± 1.92 |  |
| 2-Phenylethylamine | 0.23 | ± 0.01 | 0.83 | | ± 0.02 | *** |
| Putrescine | 33.81 | ± 1.40 | 31.51 | | ± 1.01 |  |
| Spermidine | 0.69 | ± 0.05 | 0.37 | | ± 0.05 | ** |
| Spermine | 0.65 | ± 0.06 | 0.76 | | ± 0.07 |  |
| Tryptamine | 0.37 | ± 0.01 | 0.58 | | ± 0.01 | *** |
| Tyramine | 17.40 | ± 1.11 | 23.74 | | ± 1.88 | * |
| *Volatile organic compounds* |  |  |  | |  |  |
| Acetoin | 4.87 | ± 0.07 | 4.85 | | ± 0.48 |  |
| Acetophenone | 0.08 | ± 0.01 | 0.08 | | ± 0.00 |  |
| Benzaldehyde | 0.39 | ± 0.04 | 0.10 | | ± 0.04 | *** |
| Benzyl alcohol | 0.74 | ± 0.06 | 0.20 | | ± 0.02 | ** |
| 2,3-Butanediol (RR,SS) | 132.34 | ± 7.97 | 48.95 | | ± 2.47 | ** |
| 2,3-Butanediol (RS,SR) | 320.71 | ± 19.38 | 243.35 | | ± 9.42 | ** |
| 2,3-Butanedione | 0.85 | ± 0.02 | 0.39 | | ± 0.02 | *** |
| 2-Butanone | 0.25 | ± 0.08 | 0.29 | | ± 0.16 |  |
| Dimethyl sulfone | 2.37 | ± 0.17 | 1.97 | | ± 0.05 | * |
| Phenol | 0.32 | ± 0.01 | 0.31 | | ± 0.01 |  |
| Phenyl acetaldehyde | 1.31 | ± 0.18 | 1.27 | | ± 0.02 |  |
| 2-Phenyl ethanol | 0.19 | ± 0.02 | 0.14 | | ± 0.01 |  |

**Table S5**. Animal rennet supplier, microbial growth after enrichment, and alpha diversity based on a taxonomical assessment of the bacteria at species level. Measurements were performed in triplicate. ‘+’ indicates growth for all triplicates, ‘-’ for none of the triplicates, and ‘w’ for one of the triplicates.

| Animal rennet | Commercial supplier | Microbial growth after enrichment | | Alpha diversity (species level) |  |
| --- | --- | --- | --- | --- | --- |
| R1 | B | - | 9.58 | | |
| R2 | A | + | 1.00 | | |
| R3 | A | - | 4.61 | | |
| R4 | B | + | 1.83 | | |
| R5 | A | - | 4.93 | | |
| R6 | A | - | 1.65 | | |
| R7 | B | w | 4.47 | | |

**Table S6**. Pearson correlation between brine metabolites and bacterial species (family in the case of *Babeliaceae*) found in the brines with a relative abundance above 5 %. Significant correlations are indicated in bold.

| Organic  compound | *Babe-liaceae* | *Streptococcus thermophilus* | | *Loigolacto- bacillus  rennini* | | *Weissella thailandensis* | | *Tetrageno-coccus halophilus* | |
| --- | --- | --- | --- | --- | --- | --- | --- | --- | --- |
| Acetic acid | 0.33 | 0.34 | **-0.92** | | **-0.95** | | **0.87** | |  |
| Acetoin | -0.10 | -0.09 | 0.07 | | 0.09 | | -0.06 | |  |
| Acetophenon | **0.82** | **0.83** | -0.65 | | -0.48 | | 0.34 | |  |
| Alanine | -0.09 | -0.08 | 0.33 | | 0.65 | | -0.41 | |  |
| Arginine | -0.31 | -0.31 | 0.70 | | **0.91** | | -0.69 | |  |
| Asparagine | -0.52 | -0.52 | 0.73 | | 0.59 | | -0.56 | |  |
| Aspartic acid | -0.41 | -0.41 | 0.54 | | 0.65 | | -0.46 | |  |
| Benzaldehyde | 0.53 | 0.52 | 0.58 | | 0.63 | | **-0.84** | |  |
| Benzylalcohol | 0.61 | 0.59 | 0.55 | | 0.54 | | -0.81 | |  |
| 2,3-Butanediol (RR,SS) | 0.59 | 0.57 | 0.58 | | 0.55 | | **-0.83** | |  |
| 2,3-Butanediol (RS,SR) | 0.71 | 0.70 | 0.40 | | 0.40 | | -0.70 | |  |
| 2,3-Butanedione | 0.52 | 0.50 | 0.64 | | 0.61 | | **-0.87** | |  |
| 2-Butanone | -0.44 | -0.47 | 0.19 | | 0.17 | | 0.00 | |  |
| Butyric acid | 0.55 | 0.53 | 0.60 | | 0.59 | | **-0.85** | |  |
| Cadaverine | 0.22 | 0.22 | -0.13 | | -0.42 | | 0.12 | |  |
| Citric acid | 0.47 | 0.46 | 0.67 | | 0.66 | | **-0.89** | |  |
| Citrulline | -0.36 | -0.35 | 0.12 | | -0.12 | | 0.06 | |  |
| Cysteine | -0.03 | 0.00 | 0.15 | | 0.39 | | -0.23 | |  |
| Dimethyl sulfone | **0.83** | **0.82** | 0.25 | | 0.22 | | -0.57 | |  |
| D-lactic acid | -0.31 | -0.32 | **0.98** | | **0.98** | | **-0.93** | |  |
| γ-Aminobutyric acid | -0.40 | -0.41 | **0.95** | | **0.96** | | **-0.87** | |  |
| Galactose | 0.35 | 0.33 | 0.75 | | 0.77 | | **-0.94** | |  |
| Glutamic acid | -0.22 | -0.22 | 0.68 | | **0.90** | | -0.70 | |  |
| Glutamine | -0.26 | -0.26 | 0.63 | | **0.87** | | -0.64 | |  |
| Glycine | -0.34 | -0.34 | **0.88** | | **0.95** | | **-0.83** | |  |
| Hexanoic acid | 0.57 | 0.55 | 0.56 | | 0.58 | | **-0.82** | |  |
| Hippuric acid | 0.57 | 0.55 | 0.58 | | 0.56 | | **-0.83** | |  |
| Histamine | -0.59 | -0.60 | -0.02 | | -0.23 | | 0.30 | |  |
| Histidine | -0.16 | -0.17 | **0.90** | | **0.98** | | **-0.92** | |  |
| Isobutyric acid | 0.61 | 0.60 | 0.53 | | 0.50 | | -0.79 | |  |
| Isoleucine | -0.15 | -0.16 | 0.77 | | **0.95** | | **-0.82** | |  |
| Lactic acid (D + L) | -0.43 | -0.41 | -0.58 | | -0.62 | | 0.78 | |  |
| Lactose | 0.32 | 0.30 | 0.77 | | 0.79 | | **-0.95** | |  |
| Leucine | -0.24 | -0.25 | 0.81 | | **0.97** | | **-0.82** | |  |
| L-lactic acid | -0.10 | -0.08 | **-0.85** | | **-0.88** | | **0.95** | |  |
| Lysine | -0.21 | -0.23 | 0.78 | | 0.49 | | -0.68 | |  |
| Malic acid | 0.73 | 0.75 | -0.66 | | -0.51 | | 0.39 | |  |
| Methionine | -0.30 | -0.30 | 0.77 | | **0.94** | | -0.76 | |  |
| 3-Methyl-butanoic acid | 0.56 | 0.55 | 0.59 | | 0.57 | | **-0.84** | |  |
| Ornithine | -0.08 | -0.07 | 0.35 | | 0.28 | | -0.35 | |  |
| Phenol | 0.56 | 0.55 | -0.14 | | 0.08 | | -0.11 | |  |
| Phenyl acetaldehyde | **0.94** | **0.94** | -0.62 | | -0.59 | | 0.30 | |  |
| Phenylalanine | -0.18 | -0.18 | 0.74 | | **0.93** | | -0.78 | |  |
| 2-Phenyl ethanol | **0.82** | **0.82** | 0.22 | | 0.27 | | -0.56 | |  |
| 2-Phenylethyl amine | -0.52 | -0.50 | -0.63 | | -0.63 | | **0.87** | |  |
| Proline | -0.39 | -0.39 | 0.70 | | **0.90** | | -0.66 | |  |
| Propionic acid | -0.73 | -0.71 | -0.27 | | -0.24 | | 0.55 | |  |
| Putrescine | 0.33 | 0.32 | 0.61 | | 0.42 | | -0.74 | |  |
| Serine | -0.30 | -0.32 | **0.97** | | **0.99** | | **-0.92** | |  |
| Spermidine | 0.42 | 0.40 | 0.71 | | 0.63 | | **-0.89** | |  |
| Spermine | -0.37 | -0.36 | -0.42 | | -0.59 | | 0.63 | |  |
| Succinic acid | 0.54 | 0.53 | 0.62 | | 0.58 | | **-0.86** | |  |
| Threonine | -0.22 | -0.23 | 0.80 | | **0.97** | | **-0.82** | |  |
| Tryptamine | -0.56 | -0.54 | -0.59 | | -0.60 | | **0.85** | |  |
| Tyramine | -0.57 | -0.56 | -0.50 | | -0.56 | | 0.77 | |  |
| Tyrosine | -0.49 | -0.48 | 0.09 | | 0.01 | | 0.10 | |  |
| Uric acid | 0.43 | 0.41 | 0.71 | | 0.68 | | **-0.91** | |  |
| Valeric acid | 0.01 | 0.02 | 0.29 | | 0.07 | | -0.28 | |  |
| Valine | -0.30 | -0.31 | 0.81 | | **0.97** | | -0.79 | |  |

# 2 Supplemental figures


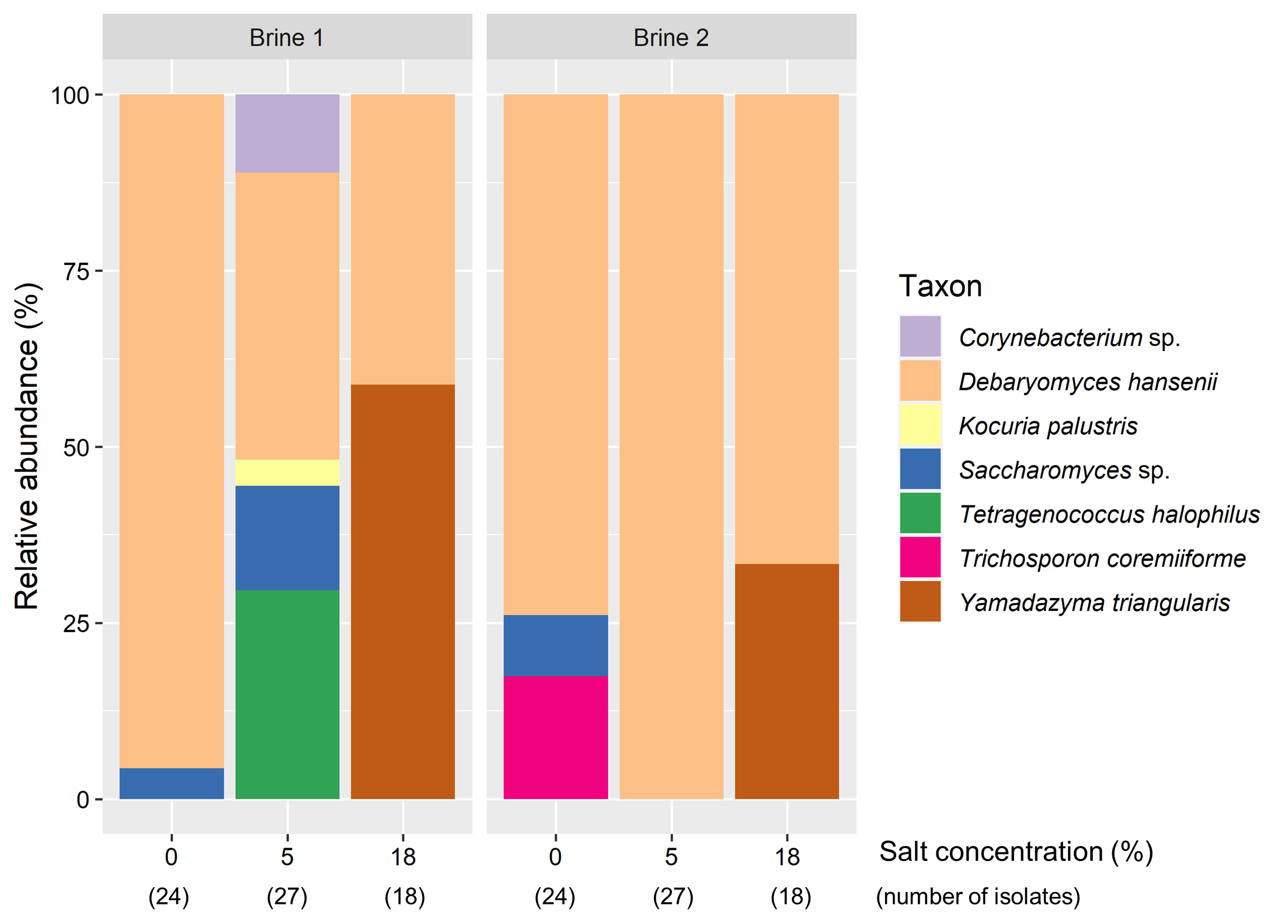
 **Figure S1**. Taxonomic assessment of the microbial isolates retrieved from plate count agar for samples from two brines of the same dairy factory. The different salt concentrations applied (0, 5, and 18 %, m/v, NaCl) are indicated on the X-axis.

**
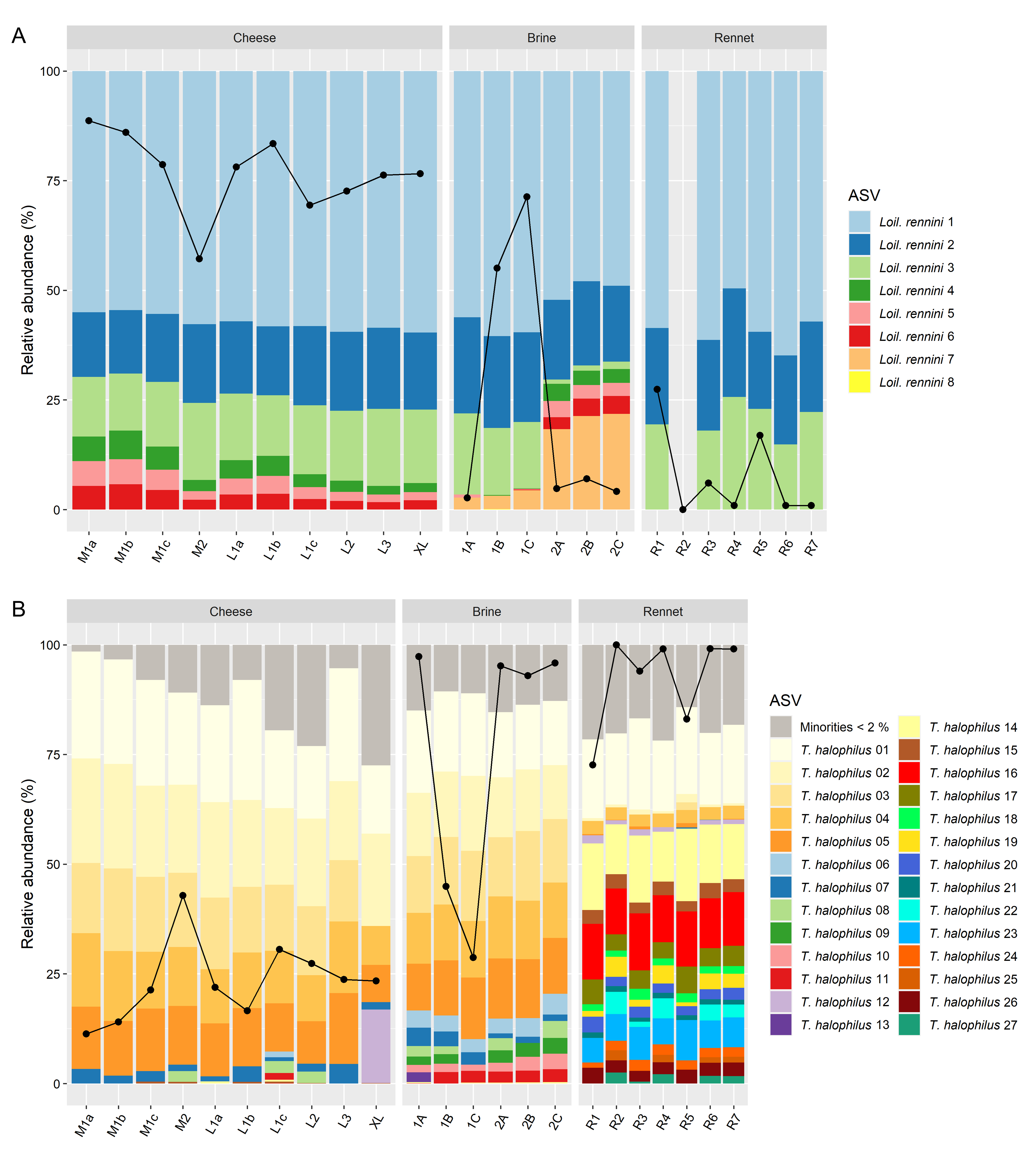
Figure S2**. Taxonomic assessment on amplicon sequence variant (ASV) level of *Loigolactobacillus rennini* (A) and *Tetragenococcus halophilus* (B)*,* based on ASVs of the full-length 16S rRNA gene, for the Gouda cheeses with an age of 31 weeks and a crack defect, the brines, and the commercial rennets. Only the ASVs from zones with cracks are shown. The black line indicates the relative abundance of the *Loil. rennini* or *T. halophilus* sequence reads in each sample.


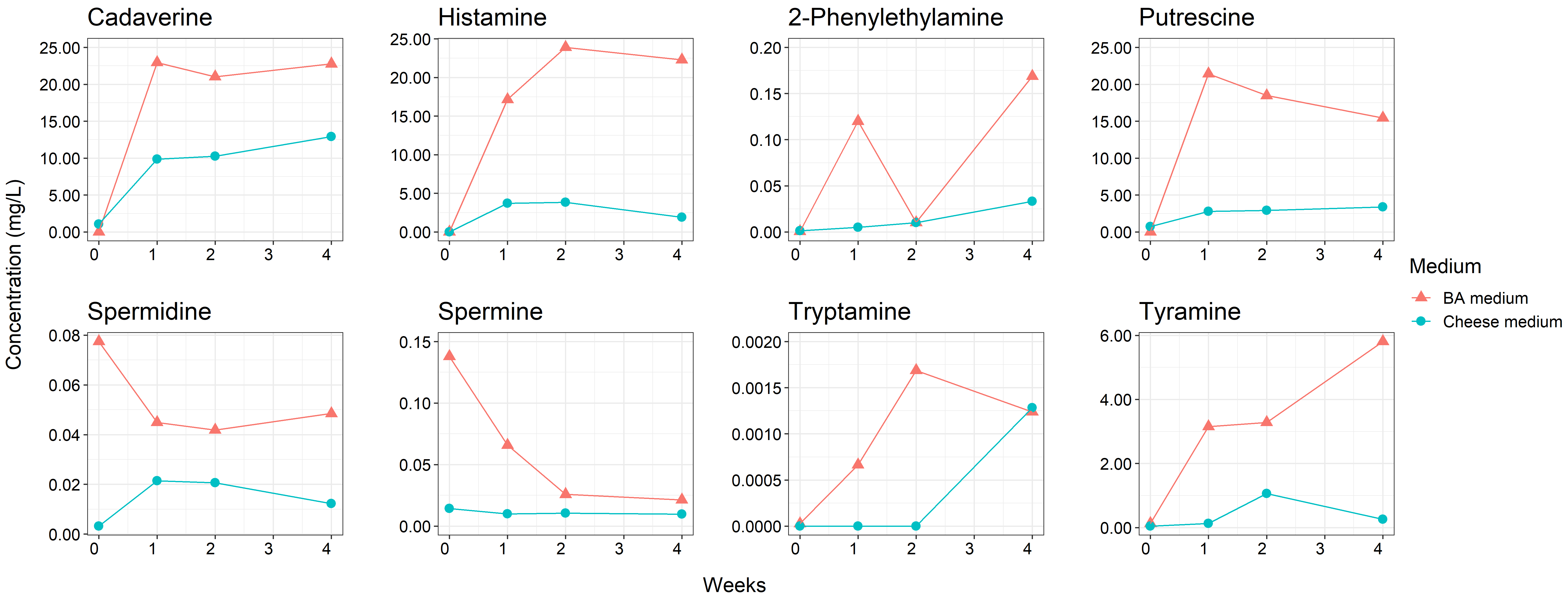


**Figure S3**. Concentrations of biogenic amines produced in a medium optimal for the production of biogenic amines (red triangles) and a cheese medium (blue circles), both inoculated with 1 % (v/v) brine (brine sample 1B), after 0, 1, 2, and 4 weeks of fermentation at 15 °C.
